# RNA-Seq-Based Transcriptome Analysis of Nitric Oxide Scavenging Response in *Neurospora crassa*

**DOI:** 10.3390/jof9100985

**Published:** 2023-10-02

**Authors:** Nan-Nan Yu, Mayura Veerana, Wirinthip Ketya, Hu-Nan Sun, Gyungsoon Park

**Affiliations:** 1 Plasma Bioscience Research Center, Department of Plasma-Bio Display, Kwangwoon University, Seoul 01897, Republic of Korea; nannan19950326@163.com (N.-N.Y.); pam7794p@gmail.com (W.K.); 2 Department of Applied Radiation and Isotopes, Faculty of Science, Kasetsart University, Bangkok 10900, Thailand; mayuraveerana@gmail.com; 3 College of Life Science and Technology, Heilongjiang Bayi Agricultural University, Daqing 163319, China; sunhunan76@163.com; 4 Department of Electrical and Biological Physics, Kwangwoon University, Seoul 01897, Republic of Korea

**Keywords:** nitric oxide, filamentous fungi, RNA sequencing, *Neurospora crassa*, vegetative growth

## Abstract

While the biological role of naturally occurring nitric oxide (NO) in filamentous fungi has been uncovered, the underlying molecular regulatory networks remain unclear. In this study, we conducted an analysis of transcriptome profiles to investigate the initial stages of understanding these NO regulatory networks in *Neurospora crassa*, a well-established model filamentous fungus. Utilizing RNA sequencing, differential gene expression screening, and various functional analyses, our findings revealed that the removal of intracellular NO resulted in the differential transcription of 424 genes. Notably, the majority of these differentially expressed genes were functionally linked to processes associated with carbohydrate and amino acid metabolism. Furthermore, our analysis highlighted the prevalence of four specific protein domains (zinc finger C2H2, PLCYc, PLCXc, and SH3) in the encoded proteins of these differentially expressed genes. Through protein–protein interaction network analysis, we identified eight hub genes with substantial interaction connectivity, with *mss-4* and *gel-3* emerging as possibly major responsive genes during NO scavenging, particularly influencing vegetative growth. Additionally, our study unveiled that NO scavenging led to the inhibition of gene transcription related to a protein complex associated with ribosome biogenesis. Overall, our investigation suggests that endogenously produced NO in *N. crassa* likely governs the transcription of genes responsible for protein complexes involved in carbohydrate and amino acid metabolism, as well as ribosomal biogenesis, ultimately impacting the growth and development of hyphae.

## 1. Introduction

Nitric oxide (NO) is a small free radical and a gaseous signaling molecule endogenously synthesized and widely conserved in eukaryotic cells. The synthesis and functions of NO have been extensively studied in mammalian and plant cells. In mammalian cells, NO is primarily synthesized by NO synthase (NOS), which regulates blood pressure, immune cytotoxicity, and neurotransmission [1]. NO activates soluble guanylate cyclase (sGC) to form cyclic guanosine monophosphate (cGMP), causing vasodilation, increasing blood supply, and lowering blood pressure [2]. NO can also stimulate the cytoplasmic motility of macrophages and regulate neutrophil adhesion and cytokine (interleukin) synthesis [3]. Moreover, NO acts as a neurotransmitter and regulates the neuronal metabolic state and dendritic spinal growth [4].

NO synthesis in plants is different from that observed in mammalian cells. There are two main modes of NO production in plant cells, namely reductive and oxidative NO production. The operation of each mode depends on the presence of oxygen in environments. In reductive NO production, cytosolic or plasma membrane-bound nitrate reductase (NR) reduces nitrate (+5) to nitrite (+3) at the expense of NAD(P)H and catalyzes a 1-electron transfer from NAD(P)H to nitrite resulting in NO (+2) formation [5]. NR is a major enzyme involved in plant nitrate assimilation, catalyzing the reduction in nitrate to nitrite. This enzyme also possesses a nitrite:NO reductase (Ni-NR) activity that can be promoted under anoxic or acidic environments. Therefore, NR-mediated NO production is usually active under anoxic or acidic conditions. Interestingly, NR can interact with nitric oxide-forming nitrite reductase (NOFNiR) belonging to the amidoxime-reducing component (ARC) protein family and reduce nitrite, producing NO [5,6,7]. In plants, the mitochondrial electron transport chain can play a role in NO production using nitrite as an alternative electron acceptor and reducing to NO under hypoxia conditions [5,6]. In addition, nitrite reduction occurs non-enzymatically in the apoplast of plant cells under low pH or highly reducing environments, producing NO [5]. In oxidative NO production, an NO synthase (NOS)-like enzyme in plants plays an important role in producing NO, in which L-arginine is oxidized to L-citrulline and NO using NADPH and O_2_ (basic reaction: L-arginine + NAD(P)H + O_2_ → L-citrulline + NAD(P) + H_2_O + NO) [5]. However, NOS in plants shows low sequence homology to the mammalian NOS. Studies have found that NO is widely involved in plant immunity, development (seed germination, flower and root development, and apical dominance), and abiotic stress, and may be related to the accumulation of salicylic acid (SA), cytokinin, auxin, and other plant hormones [5]. It has been proposed that NO may regulate auxin function by regulating the level of third messenger phosphatidic acid (PA) and negatively regulate cytokinin signaling by blunting phosphorelay activity through S-nitrosylation [5]. In addition, NO regulates the accumulation of the plant immune activator SA and the expression of SA-dependent genes through S-nitrosothiol (SNO) [5]. SA can bind and regulate the activity of many proteins, which are crucial for the establishment of plant immunity. For example, SA-binding protein 3 (SABP3) participates in negative feedback regulation of plant immunity by regulating SA binding and carbonic anhydrase activity [6]. The role of NO in plant immune function was first reported in potatoes. A study found that NO-induced the accumulation of potato phytoalexin rishitin, and this induction was weakened by adding an NO scavenger [5].

In bacteria, NO is synthesized intracellularly by NOS-like enzymes, plays a role in toxin biosynthesis, and protects against oxidative stress and ultraviolet (UV) radiation [6]. Bacterial NO protects bacterial cells against oxidative stress by reducing cellular cysteine levels, eventually activating catalase A, which is inhibited by cysteine, or enhancing superoxide dismutase A expression [7,8]. NO also reportedly protects bacterial cells from UV damage by promoting cell growth [9] and modifying antibiotics and other molecules via nitrosation [8,10].

Compared to other organisms, the biosynthesis and function of NO in fungi remain relatively less explored [11]. Endogenous NO generation has been reported in many fungi [12] and is known to regulate several morphological and physiological processes in filamentous fungi, including asexual and sexual development, spore germination, hyphal growth, infection, secondary metabolism, and enzyme production [13,14,15,16,17,18,19,20,21]. These studies suggest the potential role of NO as a major signaling molecule in the regulation of various cellular processes in filamentous fungi.

Filamentous fungi are beneficial to human life with respect to environmental conservation and are key players in industrial biotechnology [22,23]. Filamentous fungi produce biologically active secondary metabolites essential for human health [24,25,26] and industrially applicable enzymes such as cellulases, xylanases, and amylases [27,28]. Moreover, they are essential in the Earth’s material cycle, soil bioremediation, and biofuel production [22]. The identification and functional elucidation of signaling components that regulate biological processes in fungal cells are prerequisites for efficiently applying fungal resources to human life [23].

Although experimental data on NO biology in filamentous fungi are accumulating, the molecular regulatory networks of endogenous NO in fungal cells remain poorly understood. The transcriptome profiles of the three filamentous fungi (*Pleurotus eryngii*, *Shiraia* sp. S9, and *Ganoderma oregonense*) [29,30,31] demonstrated that exogenous NO upregulates genes related to heat shock stress (heat shock proteins) and heavy metal stress, as well as genes involved in secondary metabolite production in fungal cells. The transcriptome profiling of fungal cells in response to NO is a prerequisite for understanding the regulatory mechanisms of NO in filamentous fungi. However, previous studies have focused on the responses to exogenous NO, and direct responses to endogenous NO have rarely been studied. Previously, we observed that NO is endogenously generated and is involved in regulating hyphal growth, conidiation, and the transcription of cellulolytic enzymes in *Neurospora crassa* [17,21]; nonetheless, the detailed regulatory mechanisms have not been elucidated. As the first step in elucidating these regulatory mechanisms, we analyzed the transcriptome of *N. crassa* cells under endogenous NO-scavenging conditions to clarify the molecular networks that govern fungal development via endogenous NO.

## 2. Materials and Methods

### 2.1. Fungus and Culture Condition

*Neurospora crassa* strains FGSC 4200 (wild type strain ORS-SL6a, mat a), FGSC 2489 (wild type strain 74-OR23-IVA, mat A), FGSC 15509 (Δ*mss-4/NCU02295*, mat a), FGSC 12976 (Δ*gel-3/NUC08909*, mat a), and FGSC 12977 (Δ*gel-3/NUC08909*, mat A) were obtained from the Fungal Genetics Stock Center (FGSC, Manhattan, KS, USA). Fungal strains were conserved as conidia masses in silica gel at −20 °C. Fungal strains were cultured from silica stocks by placing silica gel on VM (Vogel’s minimal) agar medium (in 1 L: Na_3_Citrate 2H_2_O, 2.535 g; KH_2_PO_4_, 5 g; NH_4_NO_3_, 2 g; MgSO_4_ 7H_2_O, 0.4 g, CaCl_2_ 2H_2_O, 0.2 g; trace element solution, 200 µL; biotin solution, 200 µL; sucrose, 15 g; agar, 10 g). The agar culture flasks and tubes were incubated in a growth chamber (WiseCube incubator, Daihan Scientific, Wonju, Republic of Korea) under the condition of 30 °C in the dark for 2 days and then at 25 °C in the light (fluorescent lamps: luminous flux 1800 lm, wattage 24 W, color temperature (Kelvin) 6500 K—cool daylight) for 12 days. All gene replacement mutant strains were maintained on VM agar medium supplemented with 200 μg/mL hygromycin (Calbiochem, San Diego, CA, USA).

### 2.2. cPTIO (2-(4-carboxyphenyl)-4,5-dihydro-4,4,5,5-tetramethyl-1H-imidazolyl-1-oxy-3-oxide) Treatment and RNA Preparation for RNA Sequencing

*N. crassa* conidia were harvested from a 2-week-old culture flask. Sterile deionized (DI) water (50 mL) was added to the culture flasks and mixed vigorously. The fungal suspension was filtered through 4 layers of a sterile miracloth (Calbiochem). The filtered suspension was centrifuged at 3134× *g* for 5 min (2236R high-speed centrifuge, LaboGene, Kimpo, Republic of Korea), the supernatant was discarded, and the conidial pellet was resuspended in sterile DI water to a concentration of 10^7^ conidia/mL [17]. The conidial suspension (1 mL; 10^7^ conidia) was treated with cPTIO (10 mM final concentration in PBS) at 25 °C in the dark for 1 h [17]. The concentration of cPTIO used in this study (10 mM) was determined in our previous study in which intracellular NO level in *N. crassa* was dramatically reduced after treatment with 10 mM cPTIO [17]. Untreated conidia served as the controls. In the meantime, VM agar medium (in a 90 mm Petri dish) treated with cPTIO was prepared by spreading 100 μL of 10 mM cPTIO (cPTIO-treated group) or PBS (control group) onto the surface of VM agar. We then placed a layer of transparent cellophane sheet (Bio-Rad, Hercules, CA, USA) cut in the shape of a Petri dish on the surface of VM agar media. Then, 1 µL of conidia were placed at the center of the VM + cPTIO or VM agar covered with a cellophane sheet. All plates were incubated at 25 °C in the light for three days. After three days, fungal mycelia grown on the surface of the cellophane sheet were easily collected by scraping the cellophane surface using a spreader, and the collected fungal mycelia were stored at −80 °C. Total RNA was extracted using a TaKaRa RNAiso Plus kit (TaKaRa Bio, Tokyo, Japan), according to the manufacturer’s instructions. The RNA concentration was measured using a NanoDrop spectrophotometer (BioTek Instruments, Winooski, VT, USA).

### 2.3. RNA Sequencing

The mRNA library, cDNA synthesis, and sequencing were outsourced to Macrogen (Seoul, Republic of Korea). Total RNA from cPTIO-treated and untreated (control) samples was pretreated with DNase to remove contaminating DNA. mRNA libraries were constructed using protocols adapted from Illumina (San Diego, CA, USA). The mRNA was purified using a TruSeq Stranded mRNA LT Sample Prep Kit (Illumina), and the isolated mRNA was randomly fragmented. RNA fragments were reverse transcribed into cDNA, and sequence adapters were ligated to both ends of the cDNA fragments. The fragments were amplified, and 200–400 bp fragments were selected. Libraries were sequenced using the NovaSeq 6000 sequencing system and NovaSeq 6000 S4 Reagent Kit (Illumina). mRNA levels were assessed using fragments per kilobase of exon per million mapped fragments (FPKMs). Four libraries constructed from two replicates of each of the cPTIO-treated and untreated samples were sequenced. Gene annotation was performed using a reference sequence (*N. crassa* OR74A Genome Assembly, NC12: GCF_000182925.2_NC12).

### 2.4. Data Pre-Processing and Quality Check

Raw sequencing reads underwent initial quality checks and preprocessing at Macrogen (Seoul, Republic of Korea). Basic statistics, including total bases, reads, and GC content (%), were computed. Poor-quality data, such as sequences with adapter contamination and PCR duplicates, were removed from the original raw data. Genes with zero counts across all four samples were excluded from subsequent analysis, resulting in 8757 genes for statistical analysis, after filtering out 1831 of the 10,588 genes. Data were normalized using relative log expression (RLE) to mitigate systematic biases during sample comparisons. The gene distribution was visualized through density and box plots, utilizing raw signal data, log2-transformed signals, and RLE-normalized values (Supplementary Figure S1a–c provide additional details).

### 2.5. Screening and Functional Enrichment Analysis of DEGs (Differentially Expressed Genes)

DEGs were determined based on the fold change in expression levels (|Fc| ≥ 2) and the nbinomWald test (raw. *p* < 0.05) using DESeq2 [32]. Genes with a fold change ≥ 2 (raw. *p* < 0.05) and a fold change ≤ −2 (raw. *p* < 0.05) in cPTIO-treated samples compared to those in the CON (control) samples were defined as upregulated and downregulated genes, respectively. To investigate the relevant biological functions of the DEGs, gene ontology (GO) and protein domains were analyzed using the Database for Annotation, Visualization, and Integrated Discovery (DAVID). Molecular interactions and metabolic networks of the genes were analyzed using information from the Kyoto Encyclopedia of Genes and Genomes (KEGG) enrichment pathway (http://www.kegg.jp/kegg/pathway.html accessed on 6 August 2020). Statistical significance was set at *p* < 0.05. Screening and functional analysis of DEGs were performed by Macrogen (Macrogen).

### 2.6. Validation of DEGs by Real-Time qRT-PCR (Quantitative Reverse Transcription Polymerase Chain Reaction)

To verify the transcription of DEGs, real-time quantitative reverse transcription PCR (qRT-PCR) was performed, as previously described [33]. Briefly, total RNA isolation and cDNA synthesis were performed using RNAiso Plus (TaKaRa Bio, Shiga, Japan) and ReverTra Ace qPCR RT Master Mix with a gDNA Remover (Toyobo, Osaka, Japan), respectively, following the manufacturer’s instructions. After cDNAs were synthesized, a qPCR reaction mixture with cDNA was prepared using iQ SYBR Green Supermix (Bio-Rad, Hercules, CA, USA), according to the manufacturer’s instructions. qPCR was then performed in a thermocycler, CFX 96TM Real-Time Instrument (Bio-Rad). Twenty genes were randomly selected from the DEGs for qRT-PCR analysis. For primer design, a web program, Primer3Plus (European Molecular Biology Laboratory EMBL, Heidelberg, Germany), was used. The primer sequences are listed in Supplementary Table S1. Regarding data analysis and validation criteria, linear regression analysis was performed to compare RNA-seq and qRT-PCR results.

### 2.7. PPI Network Construction and the Identification of Hub Genes

The procedure involves gathering and preprocessing protein–protein interaction data, constructing a network (via STRING and Cytoscape), identifying central hub genes within the network (via CytoHubba), conducting functional analysis on these genes, and interpreting their biological relevance in the context of the research (via ClueGO/CluePedia). The detailed method is as follows. PPI networks were constructed using the Search Tool for the Retrieval of Interacting Genes (STRING) Version 11.5 (https://www.string-db.org/ accessed on 23 December 2022) and Cytoscape 3.9.1 software [34]. The PPI network was constructed based on information obtained from experiments and predicted from gene neighborhood, gene fusions, gene co-occurrence (with a 0.4 medium confidence score as the analysis parameter), and additional information, such as co-expression and text mining [35]. GO and pathway annotation of PPI networks were also analyzed and visualized using ClueGO version 2.5.9/CluePedia version 1.5.9 plugins from Cytoscape software [36]. The criteria for ClueGO analysis were based on a two-sided hypergeometric test with a *p* ≤ 0.05, Benjamini–Hochberg correction, and kappa score ≥ 0.4 [37]. Hub genes interacting with multiple other genes were screened using CytoHubba version 0.1, a plugin from Cytoscape software [36]. Key hub genes were identified through six topological analyses: degree, edge-percolated component (EPC), maximum neighborhood component (MNC), maximal clique centrality (MCC), closeness, and radiality [38].

### 2.8. MCODE (Molecular Complex Detection) Analysis

To identify the densely connected regions (molecular complexes) in the PPI networks, module analysis was performed using the MCODE version 2.0.2 plugin from Cytoscape version 3.9.1 (data preparation, network preprocessing, and running the MCODE algorithm through Cytoscape). In the module analysis, the cut-off degree, cut-off node score, and k-core were set to 2, 0.2, and 2.0, respectively. For evaluating and prioritizing identified complexes, we selected clusters with MCODE scores higher than 3. Functional analysis and the visualization of complexes were performed using the ClueGO version 2.5.9/CluePedia version 1.5.9 plugin with *p* < 0.05 [39,40,41].

**Figure 1:**
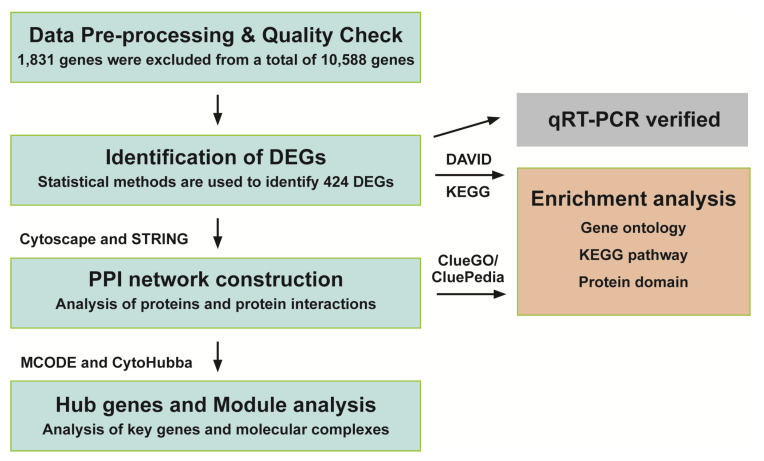
Flowchart of the proposed methodology.

In short, the RNA sequencing data were analyzed in the following order: data pre-processing and quality check, the identification of differentially expressed genes (DEGs), downstream analyses through protein–protein interaction (PPI) network construction, hub gene screening, and module analysis (Figure 1) [37,42].

### 2.9. Phenotypic Analysis of Mutants

The phenotypes of the two gene replacement mutants, Δ*mss-4* and Δ*gel-3*, were analyzed to find which functions were regulated by these genes. Three aspects during fungal development were examined: vegetative growth, asexual development, and sexual development. Δ*mss-4* and Δ*gel-3* were generated by the *Neurospora* Genome Project (http://www.dartmouth.edu/~neusporagenome), in which each target gene was replaced with a hygromycin resistance gene. We analyzed vegetative hyphal growth, aerial hyphal growth, the number of conidia formed, and the formation of protoperithecia and perithecia in these two mutants. For the control, the wild type strain (FGSC4200, ORS-SL6a, mat a) was used in phenotypic analysis. For vegetative hyphal growth, 1 µL (1 × 10^7^ conidia/mL) of conidial suspension was inoculated onto the center of VM agar plate, which was incubated at 30 °C in the dark. After 24 and 48 h, the hyphal extension on VM agar was photographed and analyzed by measuring the diameter of the hyphal extension. To investigate the growth of aerial hyphae and the formation of conidia, 2 μL of conidial suspension was inoculated onto VM agar in a glass tube (13 × 100 mm), which was incubated at 25 °C under continuous light for 7 days. The heights of the aerial hyphae were measured daily. On the 7th day, the conidia produced were harvested by adding 5 mL of deionized water to the tube, shaking the tube, and filtering the suspension through a sterile miracloth (Calbiochem). The number of conidia in the collected suspensions was counted using a hemocytometer. To analyze sexual development, wild type and mutant strains were inoculated onto SCM (Synthetic Crossing Medium) agar plates and grown at 25 °C under constant light. After 4–6 days, protoperithecia formation was examined under a stereomicroscope (SZX 16; Olympus, Tokyo, Japan). Next, the protoperithecia were fertilized by inoculating a conidial suspension of wild type strains of the opposite mating type onto the protoperithecia-formed mycelia. After 4–7 days, perithecia development was examined under a compound microscope (Olympus) and photographed using a camera (DP 73; Olympus, Tokyo, Japan). All experiments were repeated at least 3 times, and 3 replicate measurements were performed in each experiment.

### 2.10. Statistical Analysis

qRT-PCR data are presented as the mean ± standard deviation (SD) of at least nine replicates (three replicates per experiment and three independent experiments). A paired Student’s *t*-test and two-way analysis of variance were performed. A *p*-value < 0.05 was considered statistically significant. SPSS Statistics Software version 25 (IBM, Chicago, IL, USA) was used for statistical analysis.

## 3. Results

### 3.1. Identification of DEGs between cPTIO and CON (no cPTIO)

**Figure 2:**
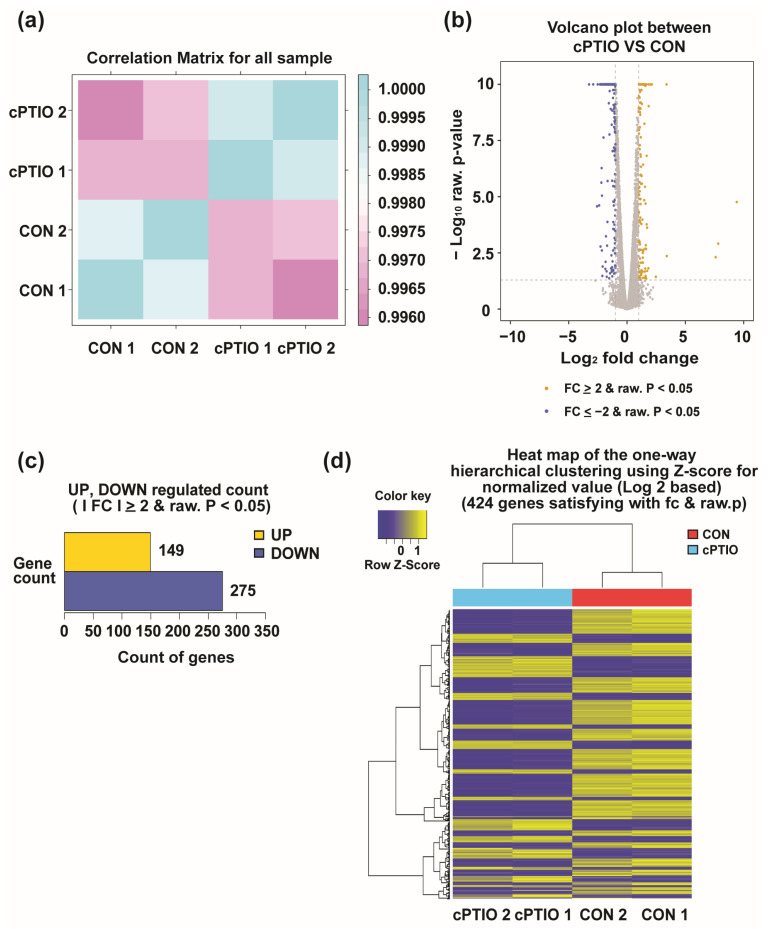
Screening for differentially expressed genes (DEGs): (**a**) Pearson correlation analysis between all biological RNA samples; (**b**,**c**) volcano plot of 424 DEGs in CON and cPTIO samples. The log_2_ fold change and the negative logarithm of the *p*-value are shown on the x- and y-axis, respectively. Down- and upregulated genes in cPTIO compared to CON samples are plotted in blue and yellow, and genes without significant differences are drawn in gray; (**d**) heatmap of 424 DEGs in CON and cPTIO samples; yellow: increased expression, blue: decreased expression. The color key indicates the intensity associated with normalized expression values. Differential expression gene analysis by DESeq2 software with raw *p* < 0.05 and FC ≥ 2 or FC ≤ −2 as the screening criteria.

RNA sequencing was performed with two replicates per treatment group (CON and cPTIO). The reproducibility of the replicates was confirmed by evaluating the similarity (Pearson’s coefficient) between samples. The closer the correlation coefficient value is to 1, the higher the similarity between samples. Our results showed that the Pearson correlation between the two biological replicates from either the CON (CON 1 and CON 2) or the cPTIO (cPTIO 1 and cPTIO 2) groups exceeded 0.9988, indicating the reliability of the RNA-seq results (Figure 2a). Differential expression profiling was performed using DESeq2 software. We found 424 DEGs between the CON and cPTIO groups among 8757 genes (Supplementary Table S2). Compared to the CON group, 149 and 275 genes were upregulated and downregulated in the cPTIO-treated group (NO scavenging), respectively (Figure 2b–d).

**Figure 3:**
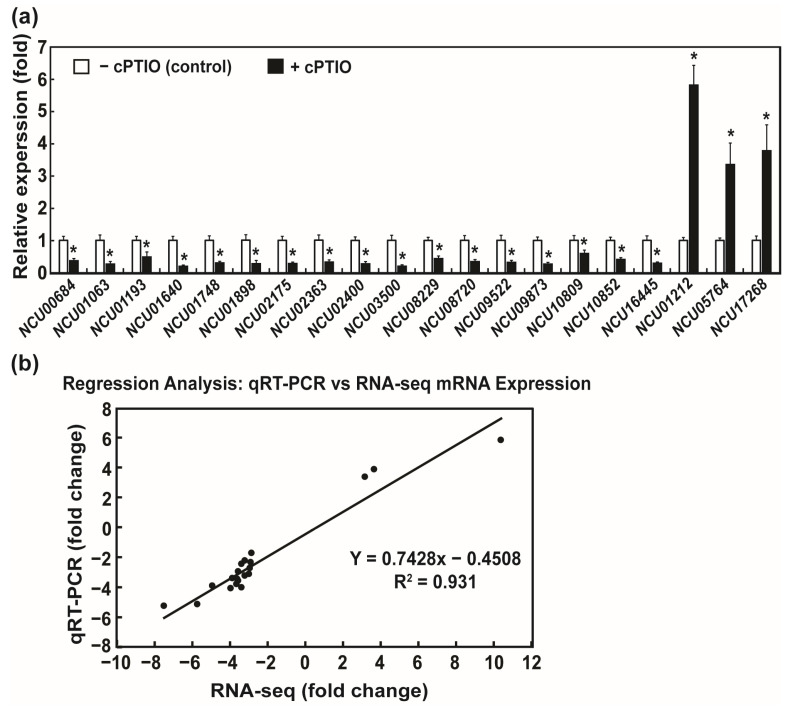
Correlation between qRT-PCR and RNA-seq results: (**a**) mRNA expression results (fold change) for twenty genes detected by qRT-PCR. Each value is the mean of nine replicate measurements (three replicates per experiment and three independent experiments). A *p*-value < 0.05 was considered statistically significant, * *p* < 0.01; (**b**) linear regression analysis between qRT-PCR and RNA-seq results for 20 genes. The fold change in the expression value (cPTIO/CON) measured by RNA-seq and qRT-PCR are shown on the x- and y-axis, respectively.

To verify the accuracy of the RNA-seq results, 20 differential genes were randomly selected for qRT-PCR. The relative transcription levels of each gene were significantly higher or lower in the cPTIO-treated group than in the CON group (Figure 3a,b). A similar trend was observed in the RNA-seq results with a significant positive correlation (R^2^ = 0.931; Figure 3b), suggesting that the RNA-seq results were reliable.

### 3.2. Functional Enrichment Analysis of DEGs

To further analyze the biological properties of the DEGs, we performed gene enrichment and functional annotation (GO) analysis of the 424 genes using DAVID. Gene ontology (GO) analysis is an international standard for gene function classification. Overall, 239 contigs were assigned to 3 main functional categories: biological processes (BPs), molecular functions (MFs), and cellular components (CCs) [43]. Nineteen GO terms were significantly enriched (*p* < 0.05; Supplementary Table S3). The eight GO terms enriched in the BP categories included response to stress (GO:0006950), carbohydrate metabolic process (GO:0005975), hexose transmembrane transport (GO:0035428), glucose import (GO:0046323), secondary metabolite biosynthetic process (GO:0044550), lipid catabolic process (GO:0016042), anion transport (GO:0006820), and transport (GO:0006810; Figure 4a). Six GO terms belonged to the following CC categories: integral component of the membrane (GO:0016021), integral component of the plasma membrane (GO:0005887), intracellular (GO:0005622), fungal-type vacuole (GO:0000324), cell wall (GO:0005618), and plasma membrane (GO:0005886; Figure 4b). In the MF category, the genes were significantly enriched in five GO terms: phosphatidylinositol phospholipase C (PLC) activity (GO:0004435), metal ion binding (GO:0046872), monooxygenase activity (GO:0004497), transmembrane transporter activity (GO:0022857), and catalytic activity (GO:0003824; Figure 4c).

KEGG pathways (http://www.kegg.jp/kegg/pathway.html accessed on 6 August 2020) are usually divided into six groups, of which only three (metabolism, genetic information processing, and cellular processes) were included in our study. Our results showed that 11 pathways were significantly enriched (*p* < 0.05; Supplementary Table S4), including metabolic pathways (ncr01100), biosynthesis of secondary metabolites (ncr01110), pentose and glucuronate interconversions (ncr00040), fructose and mannose metabolism (ncr00051), galactose metabolism (ncr00052), and amino sugar and nucleotide sugar metabolism (ncr00520), nitrogen metabolism (ncr00910), arginine and proline metabolism (ncr00330), and tyrosine metabolism (ncr00350). Ribosome biogenesis in eukaryotes (ncr03008) and endocytosis (ncr04144) were involved in genetic information processing and cellular processes, respectively (Figure 5).

Further analysis revealed four pathways related to carbohydrate metabolism (pentose and glucuronate interconversion, fructose and mannose metabolism, galactose metabolism, and amino and nucleotide sugar metabolism). Two pathways related to amino acid (arginine, proline, and tyrosine) metabolism were also identified (Figure 5). These results suggest that NO scavenging mainly affects the metabolism of carbohydrates and amino acids in fungi.

### 3.3. Enrichment of Four Protein Domains from DEGs

Protein domains are the basic units of proteins that fold, function, and evolve independently. Knowledge of protein domains is critical for understanding the biological functions of specific proteins [44]. The protein domains of the DEGs were predicted by functional annotation analysis using the InterPro and SMART databases [45]. The results showed that twelve protein domains were significantly enriched in InterPro, and four protein domains were significantly enriched in SMART (*p* < 0.05; Figure 6a,b and Supplementary Table S3). Venn diagrams, including both InterPro and SMART results, showed that four protein domains were displayed in both databases: zinc finger C2H2-type/integrase DNA-binding domain (IPR013087 and SM00355), phospholipase C, phosphatidylinositol-specific Y domain (IPR001711 and SM00149), phospholipase C, phosphatidylinositol-specific X domain (IPR000909 and SM00148), and Src homology-3 domain (IPR001452 and SM00326; Figure 6c and Table 1). Our results suggest that proteins with these four binding domains are more sensitive to intracellular NO.

**Figure 4:**
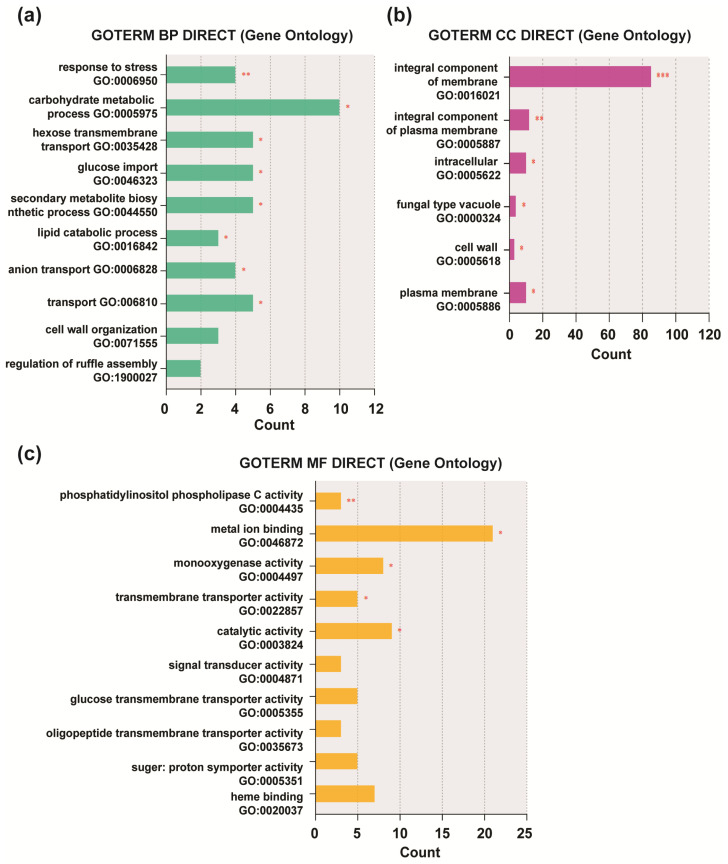
Gene ontology (GO) enrichment analysis of differentially expressed genes (DEGs) based on (**a**) biological process, (**b**) cellular component, and (**c**) molecular function. The x- and y-axis indicate the number of DEGs and the corresponding GO terms, respectively. The modified Fisher exact *p*-value < 0.05 was considered a significantly enriched GO term; * *p* < 0.05, ** *p* < 0.01, *** *p* < 0.001.

The C2H2-type zinc finger domain is a DNA-binding motif found in eukaryotic transcription factors [46]. The SH3 (Src homology 3) domain is present in many signaling proteins and mediates different processes [47]. The phospholipase C (PLCYc), phosphatidylinositol-specific (Y), and phospholipase C (PLCXc) domains are critical for the activity of phosphatidylinositol-specific phospholipase C, a eukaryotic enzyme essential for signal transduction [48]. Functional annotation analysis showed that cPTIO (NO scavengers) treatment significantly altered the expression of genes related to phosphatidylinositol phospholipase C (PLC) activity (Figure 4c). The expression (FPKM) of phosphatidylinositol phospholipase C (PLC)-encoding genes (*NCU02175*, *NCU11415*, *NCU06245*) was significantly decreased after cPTIO treatment (Figure 6d). These results indicated that NO positively regulates the expression of phosphatidylinositol phospholipase C (PLC).

### 3.4. Identification of Key Genes in the PPI (Protein–Protein Interaction) Network

To assess the PPI of DEGs between the cPTIO and CON groups, a visualized PPI network was constructed using STRING and Cytoscape software. A total of 403 nodes (proteins) and 235 edges (their interactions) were mapped onto the PPI network (Supplementary Figure S2). To identify significant interactions among proteins, we removed all single nodes, and a PPI network with 167 nodes (proteins) and 235 edges (interactions) was obtained (Figure 7a).

The top ten hub genes were filtered using the CytoHubba plugin, applying six methods. Hub genes were defined as those with high correlation in the PPI network (interacting with several other proteins) [49]. Hub genes are usually considered master switches in the network, and the loss of hub proteins is more likely to be lethal to PPI than the loss of non-hub proteins [49,50]. We found that gel-3 (*NCU08909*), mss-4 (*NCU02295*), Q7SAI0 (*NCU06969*), rho-2 (*NCU08683*), V5ILW9 (*NCU04095*), Q7S3B9 (*NCU04637*), Q7S1Y4 (*NCU07569*), and Q7S147 (*NCU09909*) were identified by more than three screening methods, and gel-3 (*NCU08909*) and mss-4 (*NCU02295*) appeared in all screening methods (Table 2). The expression levels, estimated as fragments per kilobase of exon model per million mapped fragments (FPKMs), of seven genes (*NCU08909*, *NCU02295*, *NCU06969*, *NCU08683*, *NCU04095*, *NCU04637*, *NCU07569*) were significantly reduced, whereas one gene (*NCU09909*) was upregulated following cPTIO treatment (Figure 7b). Possible interactions among the eight hub genes are shown in Figure 7c.

### 3.5. mss-4 (NCU02295) and gel-3 (NCU08909) Affect Hyphal Extension and Aerial Hyphae Development

We found that *mss-4* (phosphatidylinositol-4-phosphate 5-kinase) and *gel-3* (beta-1,3-glucanosyltransferase) were the major hub genes that showed high correlations with other proteins identified by all screening methods for analyzing the PPI network (Figure 7). To determine the role of these two genes, we analyzed the effects of deleting *mss-4* or *gel-3* in *N. crassa*. Vegetative hyphal extension on VM agar of gene replacement knockout mutants of these two genes was reduced by 4.5% (Δ*mss-4*, FGSC15509), 17.4% (Δ*gel-3*, FGSC12976), and 22.9% (Δ*gel-3*, FGSC12977) after 24 h compared to the wild type, respectively (Figure 8a,b and Supplementary Figure S3a,d). The reduction in hyphal growth was more pronounced after 48 h. Mycelial density was lower in Δ*mss-4* and Δ*gel-3* than in the wild type, indicating retarded vegetative growth due to *mss-4* or *gel-3* deletion (Supplementary Figure S3b,c).

We also measured the height of the aerial hyphae and the number of conidia formed. Compared with the wild type, the *gel-3* mutant showed a significant reduction in aerial hyphal height on both days 1 and 2, while the *mss-4* mutant showed a reduction only on day 2. However, the difference between the wild type and mutants was not significant after 3 days (Figure 9a,b and Supplementary Figure S4). The total number of conidia formed after 7 days did not significantly differ between the wild type and all mutants (Figure 9c). Hyphal elongation and branching, aerial hyphae formation, and conidial production are important processes in the asexual development of *N. crassa* [51].

Sexual development of wild type and mutant strains was also analyzed. We found that all mutants (Δ*mss-4* and Δ*gel-3*) were able to form protoperithecia and perithecia normally (Supplementary Figure S5). Based on microscopic observation, the *gel-3* mutant showed a reduction in perithecia formation compared to the wild type (Supplementary Figure S5). However, quantification for verifying this may be needed in future research.

### 3.6. GO and KEGG Analyses of the PPI Network

To elucidate the regulatory mechanisms by which *mss-4* and *gel-3* promote hyphal growth, we explored the related biological processes (BPs) and molecular functions (MFs) of *mss-4* and *gel-3* in the PPI network. First, we visualized the PPI complex network using GO and KEGG analyses. The enriched functional terms were visualized using the ClueGo/CluePedia plugin in Cytoscape [52,53]. The results showed that 11 terms (functional categories) were significantly enriched, including phospholipid binding (GO:0005543), phosphatidylinositol metabolic process (GO:0046488), phosphatidylinositol-mediated signaling (GO:0048015), carboxylic acid transmembrane transport (GO:1905039), fungal-type cell wall polysaccharide biosynthetic process (GO:0051278), carboxy-lyase activity (GO:0016831), carbohydrate metabolic process (GO:0005975), small molecule catabolic process (GO:0044282), pentose and glucuronate interconversions (KEGG:00040), putrescine biosynthetic process (GO:0009446), and nitrogen metabolism (KEGG:00910; Figure 10). Notably, *mss-4* (*NCU02295*) was mainly involved in phosphatidylinositol metabolic processes (GO:0046488), whereas *gel-3 (NCU08909*) was mainly involved in carbohydrate metabolism (GO:0005975) and fungal cell wall polysaccharide biosynthesis (GO:0051278; Supplementary Figure S6). Therefore, we raise the possibility that *mss-4* and *gel-3* may be involved in NO-regulated fungal growth through the above-mentioned processes.

### 3.7. NO may Regulate Ribosome Biogenesis through a Molecular Complex

**Figure 5:**
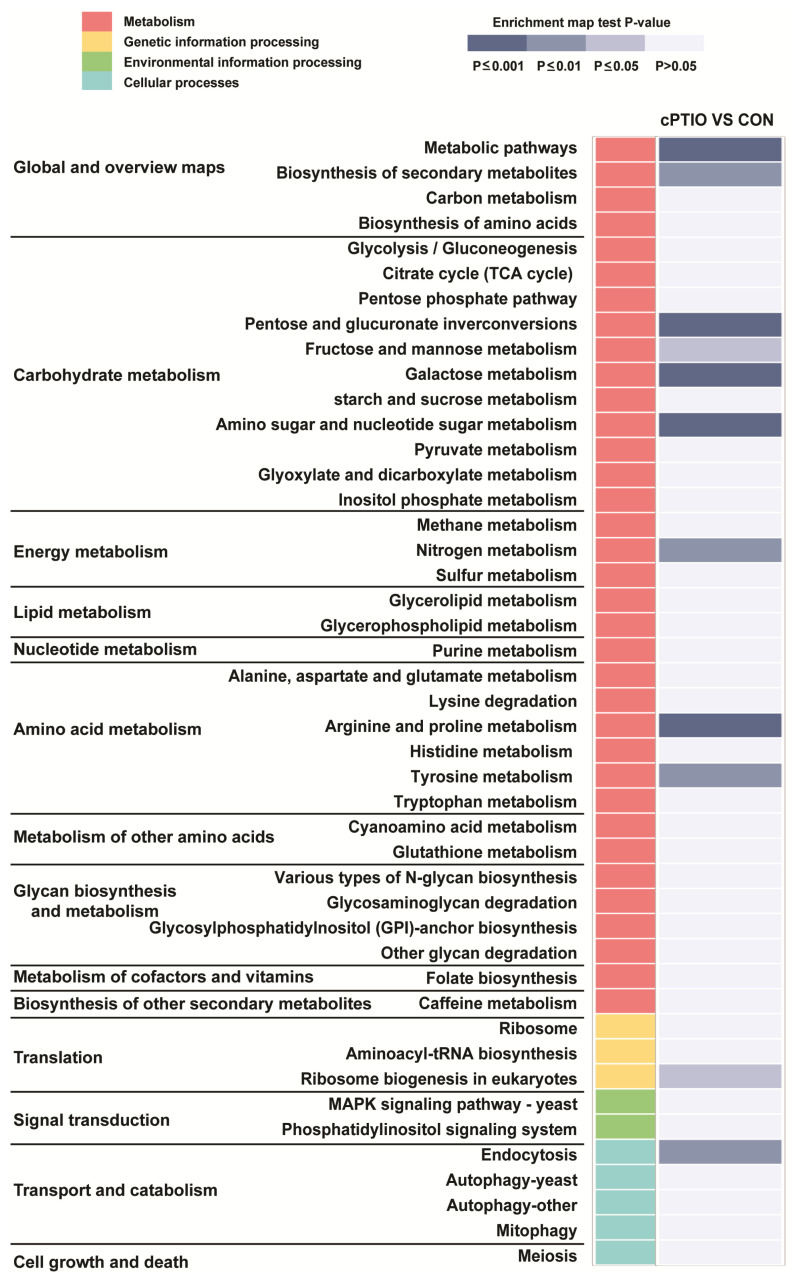
KEGG pathway enrichment analysis of differentially expressed genes (DEGs). The heatmap shows the list of pathways regulated in cPTIO vs. CON, and 11 significant enrichment pathways involved in metabolism (red), genetic information processing (yellow), and cellular processes (cyan), respectively. The enrichment map is colored according to the gradient level of the *p*-value (*p* ≤ 0.001 to > 0.05). Light gray indicates no significant difference (*p* > 0.05).

**Figure 6:**
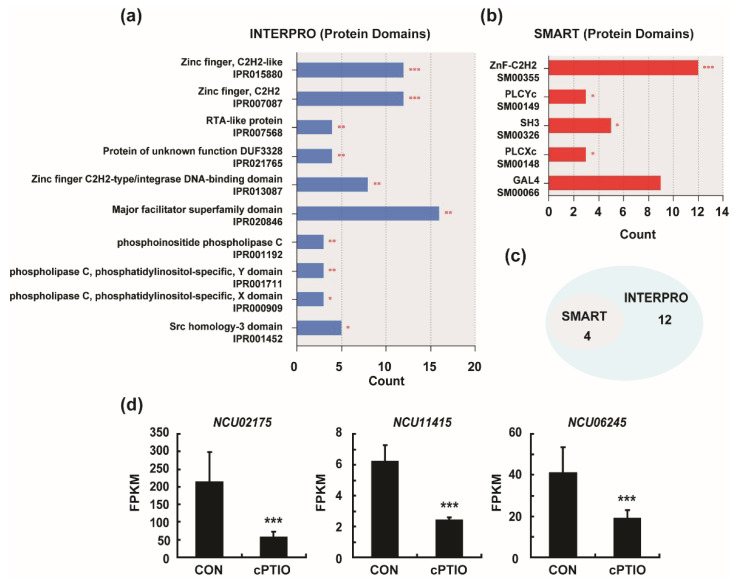
InterPro and SMART protein domain analysis of all DEGs: (**a**) the top ten InterPro protein domains; (**b**) the top five SMART protein domains; (**c**) Venn diagram for InterPro protein domains and SMART protein domains; (**d**) fragments per kilobase of exon model per million mapped fragments (FPKMs) of three phosphatidylinositol phospholipase C-encoding genes obtained by RNA sequencing analysis. Enrichment analysis of protein domains by the DAVID tool (DomainCharts modules). The x-axis shows the number of DEGs, and the y-axis indicates the corresponding protein domains; terms with *p* < 0.05 were considered significantly enriched; * *p* < 0.05, ** *p* < 0.01, *** *p* < 0.001.

**Figure 7:**
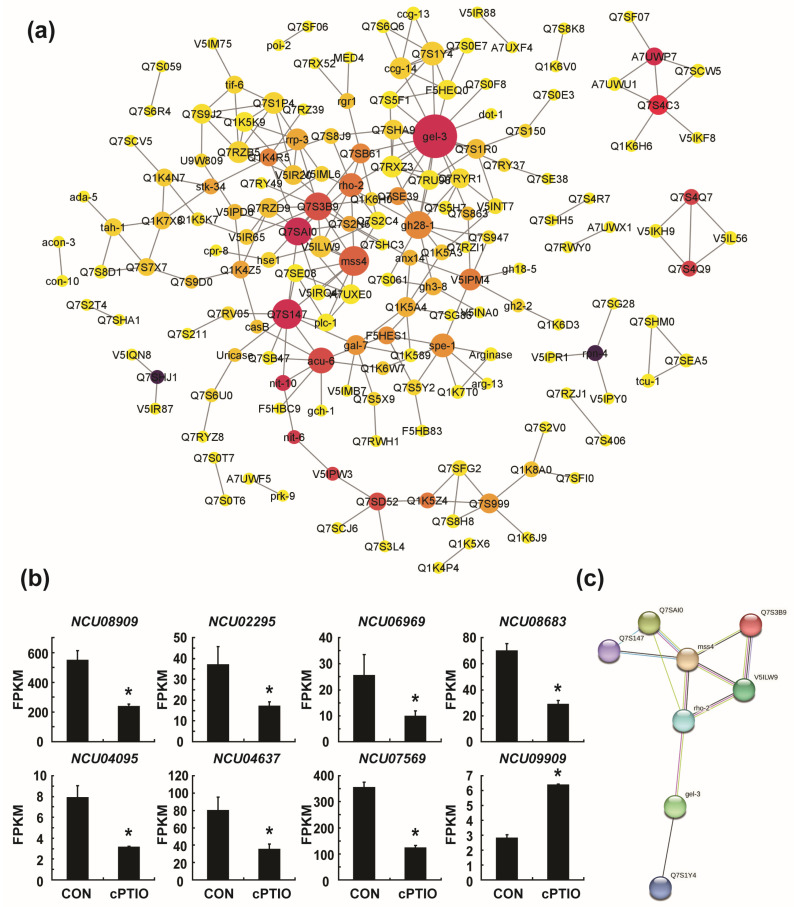
Hub gene identified from the PPI networks: (**a**) the protein–protein interaction (PPI) network was constructed by STRING and Cytoscape. The node size represents the degree of connectivity, and the node color represents the change in the betweenness centrality value; (**b**) fragments per kilobase of exon model per million mapped fragments (FPKMs) of the eight hub genes obtained by RNA sequencing analysis. A *p*-value < 0.05 was considered statistically significant; * *p* < 0.01; (**c**) PPI network of the eight hub genes.

**Figure 8:**
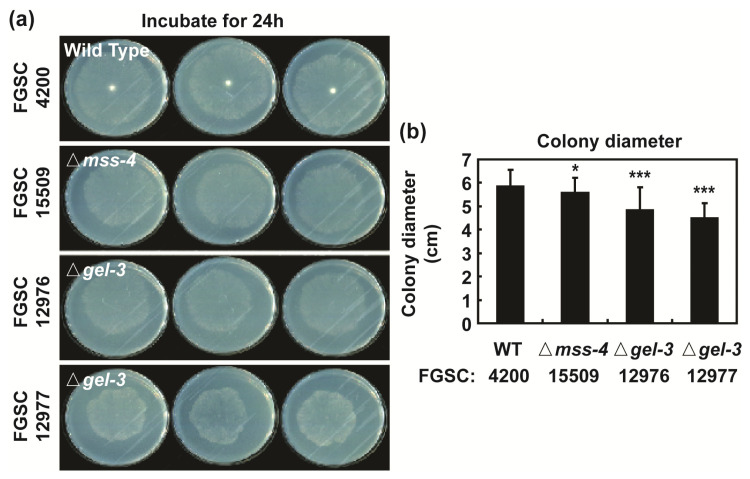
Basal hyphal growth of *N. crassa*: hyphal formation of wild type and Δ*mss-4*/FGSC15509, Δ*gel-3*/FGSC12976, and Δ*gel-3*/FGSC12977 strains on VM agar plates after 24 h (**a**); colony diameters of growing cultures were measured after 24 h (**b**). Each value is the mean of twelve replicate measurements (three replicates per experiment and four independent experiments). A *p*-value < 0.05 was considered statistically significant; * *p* < 0.05, *** *p* < 0.001.

**Figure 9:**
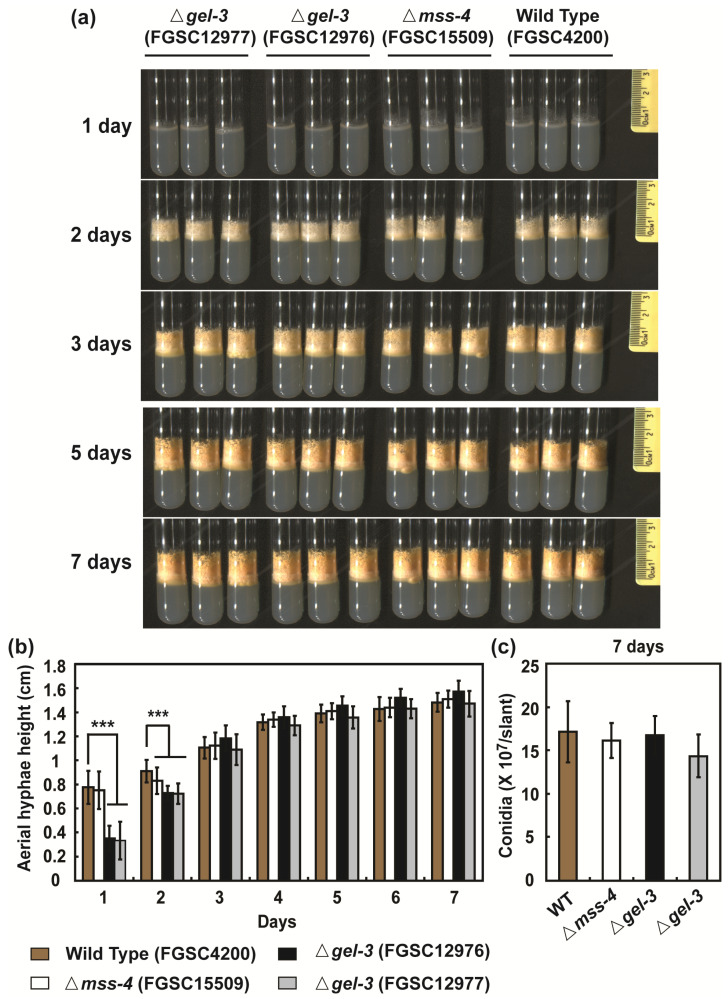
Aerial hyphae growth and conidia production in *N. crassa*: (**a**) images of all strains grown (wild type/FGSC4200, Δ*mss-4*/FGSC15509, Δ*gel-3*/FGSC12976, and Δ*gel-3*/FGSC12977) in glass tubes containing VM agar medium for indicated times; (**b**) the height of aerial hyphae were recorded from 1 day to 7 days; (**c**) conidia were collected on the seventh day, and the number of conidia per tube was counted. Each value is the mean of twelve replicate measurements (three replicates per experiment and four independent experiments). A *p*-value < 0.05 was considered statistically significant; *** *p* < 0.001.

**Figure 11:**
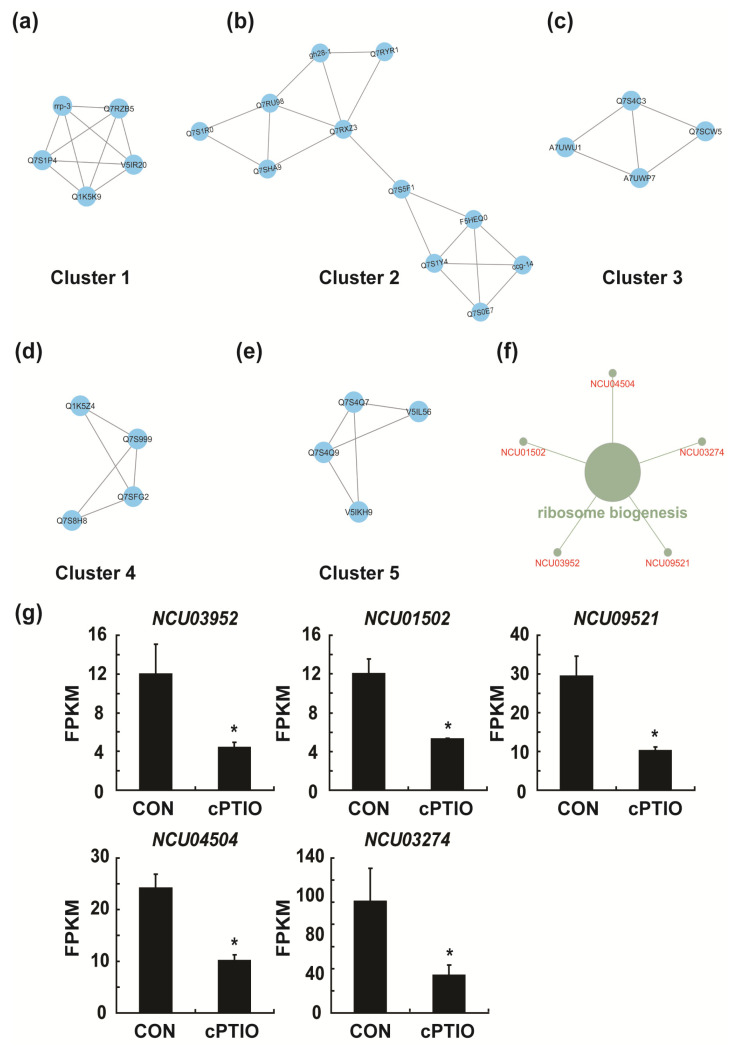
The top five clusters selected from the PPI network by the MCODE plugin: (**a**) cluster 1, score = 5; (**b**) cluster 2, score = 3.6; (**c**) cluster 3, score = 3.333; (**d**) cluster 4, score = 3.333; (**e**) cluster 5, score = 3.333; (**f**) function analysis of cluster 1; (**g**) fragments per kilobase of the exon model per million mapped fragments (FPKMs) of five genes obtained by RNA sequencing analysis; a *p*-value < 0.05 was considered statistically significant, * *p* < 0.01.

Because various fundamental biological functions are often performed through protein complexes [54], analysis of protein complexes is important to elucidate the functions involved in PPI networks. Therefore, the possibility of forming protein complexes was analyzed using the MCODE plugin. We detected highly interconnected regions (which may represent molecular complexes) in large PPI networks. However, there were five clusters (modules) with an MCODE score higher than three (one cluster with a score higher than five; Figure 11a–e). The highest-scoring clusters were Q7RZB5 (*NCU03952*), Q1K5K9 (*NCU01502*), Q7S1P4 (*NCU09521*), rrp-3 (*NCU04504*), and V5IR20 (*NCU03274*; Figure 11f); the proteins encoded by these five genes were likely associated with each other to form protein complexes. Moreover, the highest-scoring cluster was associated with ribosome biogenesis in functional gene analysis of the clusters (GO:0042254; Figure 11f). Ribosome biogenesis was one of the major functions affected by cPTIO treatment in KEGG analysis (Figure 5). The expression (fragments per kilobase of exon model per million mapped fragments) of these five genes was significantly decreased after cPTIO treatment (Figure 11g), suggesting the involvement of NO in regulating ribosome biogenesis.

## 4. Discussion

Our transcriptome analysis showed that endogenous NO regulated the transcription of 4.84% (424 DEGs/8757 total genes × 100%) of *N. crassa* genes. Among these genes, the transcription of about 70% of genes was downregulated in the absence of endogenous NO. As demonstrated in several filamentous fungi, exogenous NO modulates the transcription of genes involved in stress control and secondary metabolite production [29,30,31]. However, molecular data regarding the direct effects of endogenously generated NO are limited. Our transcriptional data regarding endogenous NO removal may play a pioneering role in elucidating the molecular regulatory mechanisms of endogenous NO in fungal development and physiology.

GO, KEGG pathway, and PPI network analyses showed that endogenous NO regulates the transcription of carbohydrate metabolism-related genes. Carbohydrate metabolism is a fundamental biochemical process that ensures a continuous energy supply to living cells [55]. In our data, the transcription of genes related to the four pathways of carbohydrate metabolism, pentose and glucuronate interconversion, fructose and mannose metabolism, galactose metabolism, and amino sugar and nucleotide sugar metabolism was significantly affected by endogenous NO. NO is essential for fungal asexual and sexual development, as well as the production of secondary metabolites [12], all of which are energy-demanding processes. Therefore, a high level of carbohydrate metabolism controlled by intracellular NO may be essential for acquiring sufficient energy for fungal biological processes.

The association between intracellular NO and the energy-producing metabolism also explains our observation that intracellular NO is related to hyphal growth in *N. crassa*. Our previous study found that NO scavenger (cPTIO) treatment retarded hyphal growth and slowed conidia formation [17]. In contrast, the addition of exogenous NO (SNP and AEMP3) increased basal hyphal growth and conidia formation [17]. Interestingly, we also found no changes in NO levels in the knockout mutants of NOS-like genes (*NCU01086*, *NCU04077*, *NCU05006*, and *NCU05185*) in *N. crassa*, and the knockout mutants of these genes did not show any significant changes in vegetative growth and conidiation, showing similar phenotypes to the wild type [17]. This might imply that NOS (nitric oxide synthase) is not important in *N. crassa* NO synthesis [29,30,31]. Therefore, the intracellular NO synthesis pathway still requires further study in *N. crassa*.

Two genes, namely, *gel-3* and *mss-4*, identified in the present study as hub genes in the molecular networks regulated by intracellular NO, are known to be involved in spore germination and hyphal growth in *N. crassa* [56,57]. *gel-3* encodes β-(1,3)-glucanosyltransferase in *N. crassa* and is strongly expressed in germinating conidia and elongating hyphae [57]. *mss-4* is an essential gene whose deletion results in altered hyphal morphology and aberrant branching [56]. Similarly, in our results, we also found that Δ*mss-4* and Δ*gel-3* mutant strains had slow vegetative growth and aerial hyphal growth. Defects in growth shown in *gel-3* and *mss-4* mutants resemble those observed in *N. crassa* after cPTIO treatment. We previously observed hyphal growth retardation after cPTIO treatment [17]. These results demonstrate a possibility that endogenous NO may be involved in the regulation of fungal vegetative growth through controlling the expression of *gel-3* and *mss-4* or other mechanisms in *N. crassa*. Spore germination and hyphal growth are essential developmental processes requiring a continuous supply of energy and building blocks. Scavenging of endogenous NO modulates the expression of genes involved in carbohydrate and amino acid metabolism, phosphatidylinositol metabolism, and ribosome biogenesis, which may be related to generating energy and building blocks. Ribosomal biogenesis in eukaryotes involves producing and correctly assembling four rRNAs and approximately eighty ribosomal proteins. Without these proteins, ribosome biogenesis is stalled and cell growth is halted, even under optimal growth conditions [58]. During fungal hyphal growth, active protein synthesis occurs, and ribosomal biogenesis is essential. However, we do not have any direct evidence showing the interaction between endogenous NO and *gel-3* and *mss-4* in the regulation of fungal vegetative growth in this study. Therefore, it is immature to speculate about the relationship between endogenous NO and *gel-3* and *mss-4*, which still requires further verification in the future.

Our results also showed that NO scavenging modulated phosphatidylinositol phospholipase C (PLC) activity, and protein domains such as phospholipase C, phosphatidylinositol-specific, Y domain phospholipase C, phosphatidylinositol-specific, and X domain were significantly enriched in the DEGs under NO scavenging. The expression levels (FPKM) of PLC-encoding genes (*NCU02175*, *NCU11415*, *NCU06245*) were positively regulated by endogenous NO. Overall, our results suggest that endogenous NO is involved in phospholipase C-mediated cellular regulation. Phosphoinositide-specific phospholipase C catalyzes the hydrolysis of phosphatidylinositol 4,5-bisphosphate (PIP2), producing two secondary messengers, inositol 1, 4, 5-trisphosphate (IP3), which induces Ca^2+^ release from intracellular Ca^2+^ stores, and diacylglycerol (DAG), which binds to the Ca^2+^-dependent C2 domain and activates PKC [48]. A previous study demonstrated that PLC is involved in hyphal elongation by regulating intracellular Ca^2+^ levels in *N. crassa* [59]. Therefore, our data suggest that endogenous NO governs intracellular Ca^2+^ accumulation by upregulating PLC expression.

Interestingly, our data demonstrate that NO also influences the biosynthesis of secondary metabolites in *N. crassa*. Functional enrichment analysis of all the DEGs revealed that NO scavenging significantly affected secondary metabolite biosynthesis (GO:0044550 and ncr01110). Studies have found that NO fumigation stimulates the accumulation of flavonoids and phenolic compounds in mushrooms [60]. LPS (lipopolysaccharides; activate NO production) treatment activates pseudoflavin A1 and A2 biosynthesis in *Penicillium* sp. and significantly accelerates shornephine and novaquinone production in *Aspergillus* sp. [61]. Notably, the secondary metabolites produced by filamentous fungi have numerous industrial applications. Our results suggest that endogenous NO strongly regulates secondary metabolite synthesis, providing a new direction for future studies.

Our results also showed that the expression levels of gene-encoding proteins with zinc finger C2H2-type/integrase DNA-binding domains were significantly modified by NO scavenging. Previously, we demonstrated that endogenous NO regulates the transcription of cellulolytic enzyme genes in *N. crassa* [21]. Cellulase transcription factors usually contain zinc finger C2H2-type protein domains [62], suggesting that NO regulates the transcription of cellulase-encoding genes by modulating the expression of zinc finger-type transcription factors. Filamentous fungi secrete large amounts of extracellular enzymes that can be used in industry [23]. Elucidation of the NO-mediated regulatory mechanisms of cellulase production may be necessary to mass-produce industrial enzymes of fungal origin.

## 5. Conclusions

The conclusion of the study suggests that endogenous nitric oxide (NO) in *N. crassa* regulates various cellular processes, including mycelial growth, metabolism, and ribosome biogenesis. Specific genes, like *gel-3* and *mss-4*, may possibly play crucial roles in this regulation, although their molecular interactions with NO require further exploration. The hypothesis proposes that NO positively influences phospholipase C (PLC) activity, possibly through Ca^2+^ signaling. While this study focused on transcriptome analysis, it sets the stage for future research on the broader biological implications of endogenous NO in filamentous fungi.

## Figures and Tables

**Figure 10:**
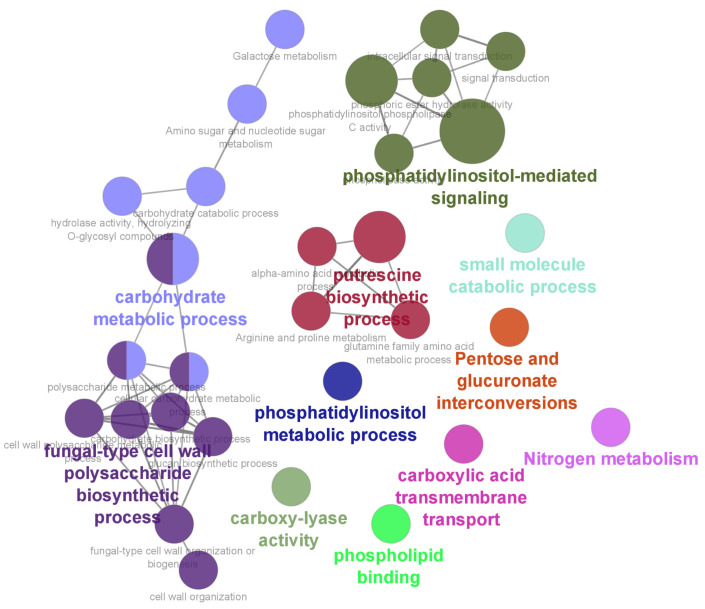
Functional enrichment terms were visualized by the ClueGO/CluePedia plugin. A *p* ≤ 0.05, Benjamini–Hochberg correction, and kappa score ≥ 0.4 were used as criteria for the two-sided hypergeometric test in ClueGO enrichment analysis. Functional enrichment analyses were inferred from a PPI network of 167 genes. The size and color of the node represent the *p*-values and various molecular functions and biological processes. The same color represents items that represent a functional group. The maximum node (minimum *p*-value) in the same functional group is shown in bold and represents the most enriched term.

**Table 1:** Protein domain analysis from InterPro and SMART databases.

Protein Domain	InterPro Count *p*-Value	SMART Count *p*-Value	Gene ID
Zinc finger C2H2 ^a^	8 0.0026	12 0.0001	*NCU03878*, *NCU05022*, *NCU09333*, *NCU03552*, *NCU04848*, *NCU00694*, *NCU06186*, *NCU01629*
PLCYc ^b^	3 0.0098	3 0.0103	*NCU02175*, *NCU11415*, *NCU06245*
SH3 ^c^	5 0.0160	5 0.0129	*NCU03888*, *NCU01883*, *NCU04095*, *NCU04841*, *NCU04637*
PLCXc ^d^	3 0.0158	3 0.0168	*NCU02175*, *NCU11415*, *NCU06245*

^a^: Zinc finger C2H2-type/integrase DNA-binding domain; ^b^: phospholipase C, phosphatidylinositol-specific, Y domain; ^c^: Src homology-3 domain; ^d^: phospholipase C; phosphatidylinositol-specific, X domain.

**Table 2:** Top 10 hub genes identified in the CytoHubba plugin.

Category ^a^	MCC ^b^	MNC ^c^	Degree ^d^	EPC ^e^	Closeness	Radiality
Gene top 10	***NCU08909*** ^a^	***NCU08909***	***NCU08909***	***NCU02295***	***NCU08909***	*NCU06969*
	*NCU09521*	***NCU02295***	*NCU09909*	*NCU08683*	*NCU06969*	***NCU02295***
	*NCU03952*	*NCU07569*	***NCU02295***	*NCU06969*	***NCU02295***	*NCU07569*
	*NCU01502*	*NCU08683*	*NCU04637*	***NCU08909***	*NCU09909*	*NCU08683*
	***NCU02295***	*NCU04095*	*NCU02369*	*NCU04095*	*NCU08683*	*NCU06252*
	*NCU04504*	*NCU00451*	*NCU06969*	*NCU07569*	*NCU04637*	*NCU04637*
	*NCU03274*	*NCU09521*	*NCU09873*	*NCU02369*	*NCU06252*	***NCU08909***
	*NCU07569*	*NCU03952*	*NCU08683*	*NCU00451*	*NCU02369*	*NCU04095*
	*NCU00451*	*NCU07787*	*NCU07569*	*NCU04637*	*NCU02137*	*NCU02137*
	*NCU03950*	*NCU06969*	*NCU01271*	*NCU06252*	*NCU04095*	*NCU09873*

^a^: Bold gene symbols indicate overlapping hub genes among the top ten genes ranked by six methods; ^b^: maximal clique centrality; ^c^: maximum neighborhood component; ^d^: node connect degree; ^e^: edge-percolated component [38].

## Data Availability

Not applicable.

## Supplementary Materials

The following supporting information can be downloaded at https://www.mdpi.com/article/10.3390/jof9100985/s1. Figure S1: Quality checking of RNA-seq data; Figure S2: The protein–protein interaction (PPI) network constructed using STRING Version 11.5; Figure S3: Analysis of basal hyphal growth of *N. crassa*; Figure S4: Analysis of aerial growth and conidia production of *N. crassa*; Figure S5: Analysis of perithecia formation in *N. crassa*; Figure S6: GO terms and KEGG pathway visualized using the ClueGO/CluePedia plugin from Cytoscape; Table S1: Primer sequences for 20 genes and 18s rRNA (reference gene); Table S2: A total of 424 differentially expressed genes (DEGs) between control (no cPTIO) and cPTIO-treated groups; Table S3: Analysis for gene ontology (GO) and protein domains using DAVID; Table S4: KEGG pathway enrichment analysis.
