# Supplementary material for: RNA-Seq-Based Transcriptome Analysis of Nitric Oxide Scavenging Response in Neurospora crassa
Source: J Fungi (Basel). 2023 Oct 2;9(10):985. doi: 10.3390/jof9100985 (PMC10607626; doi:10.3390/jof9100985)
Supplement: Supplementary file 1 [file jof-09-00985-s001.zip › 20230917 Supplementary figures R1.pdf]

Supplementary Information

# RNA-Seq-Based Transcriptome Analysis of Nitric Oxide Scavenging Response in *Neurospora crassa*

**Nan-Nan Yu [1,†], Mayura Veerana [2,†], Wirinthip Ketya [1], Hu-Nan Sun [3] and Gyungsoon Park [1,4,*]**

[1] Plasma Bioscience Research Center, Department of Plasma-Bio Display, Kwangwoon University, Seoul 01897, Republic of Korea; nannan19950326@163.com (N.-N.Y.); pam7794p@gmail.com (W.K.)
[2] Department of Applied Radiation and Isotopes, Faculty of Science, Kasetsart University, Bangkok 10900, Thailand; mayuraveerana@gmail.com
[3] College of Life Science and Technology, Heilongjiang Bayi Agricultural University, Daqing 163319, China; sunhunan76@163.com
[4] Department of Electrical and Biological Physics, Kwangwoon University, Seoul 01897, Republic of Korea
* Correspondence: gyungp@kw.ac.kr; Tel.: +82-2-940-8324
† These authors contributed equally to this work.

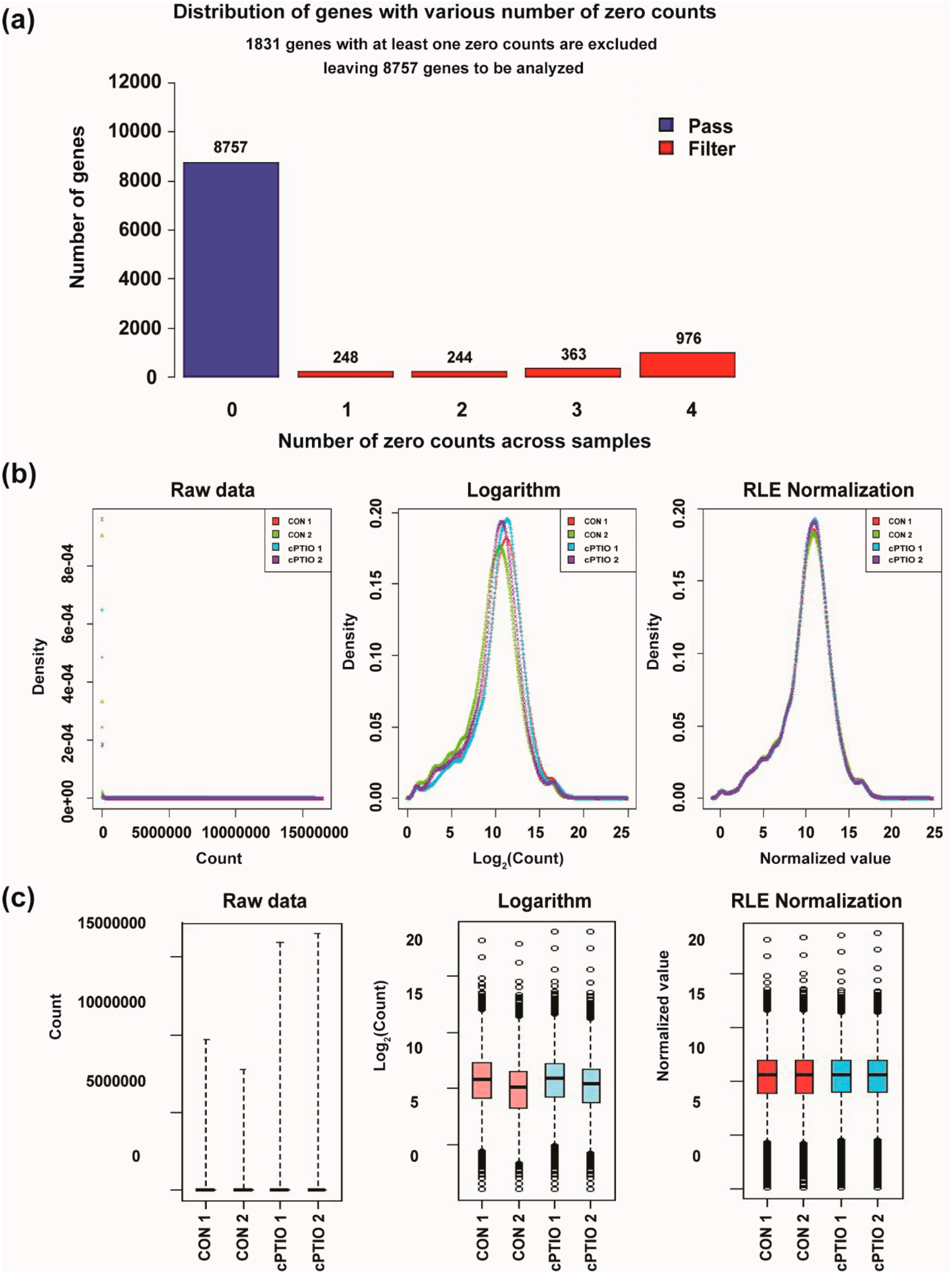


**Supplementary Figure S1. Quality checking of RNA-seq data**. (a) Number of zero counts across samples. A total of 1831 genes with at least one count value of 0 in 4 samples were filtered out and leaving 8,757 genes were counted for analysis. (b and c) Density plot (b) and

box plots (c) of normalized data using Relative Log Expression (RLE) normalization method based on raw signal (count) and logarithm (based 2). A line in the middle of each box column represents the median of data, and the top and bottom of box column correspond to the 75th and 25th percentiles of data, respectively. Whiskers show the maximum and minimum values, excluding outliers (circles).

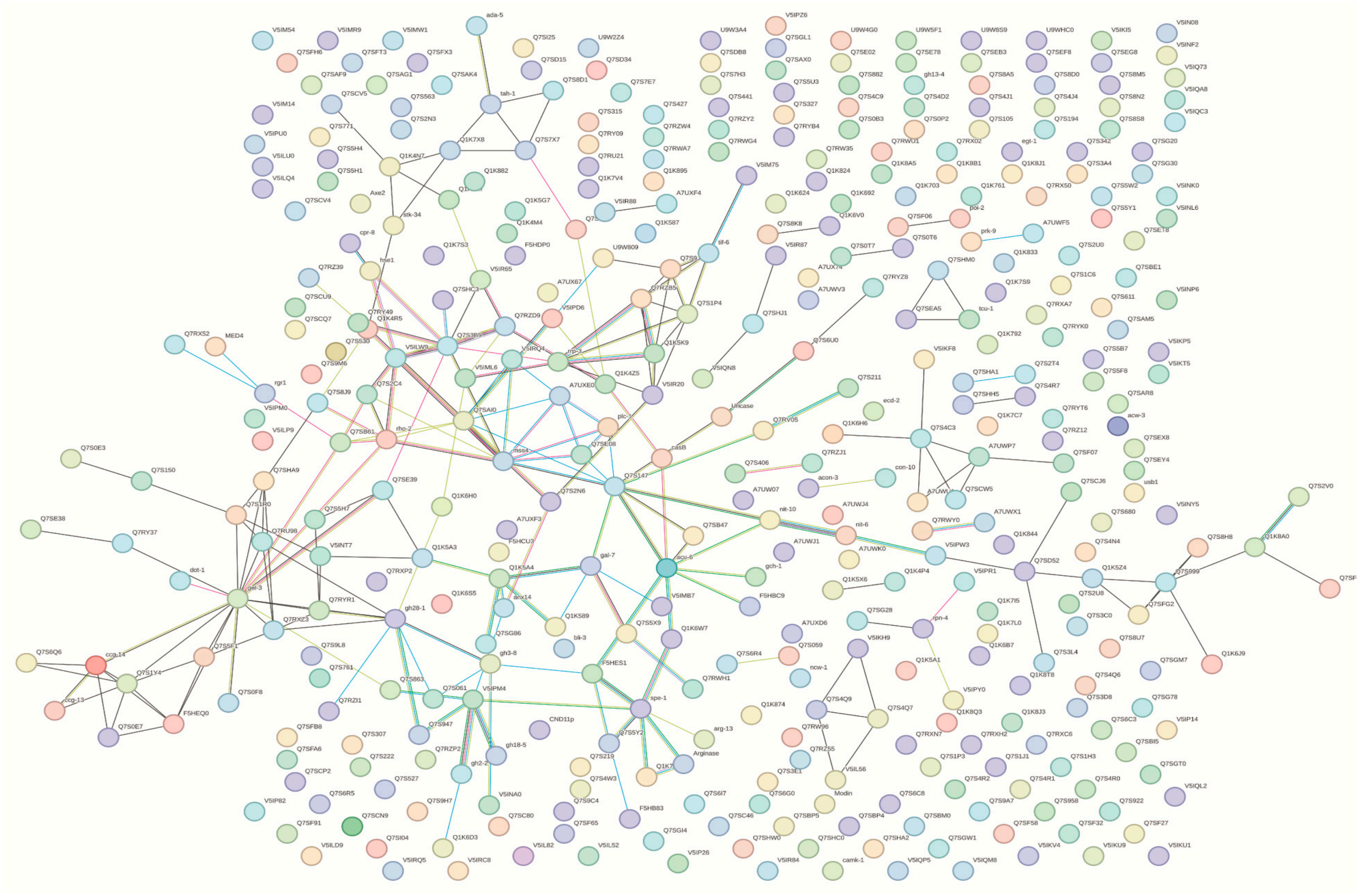

**Supplementary Figure S2. The protein-protein interaction (PPI) network constructed using STRING version 11.5.** The PPI network of all differentially expressed genes includes 235 edges (interaction) among 403 nodes (protein). Each circle and line represent protein and connectivity, respectively. Different color indicates; purple: experiments, blue: co-occurrence, yellow: text mining, light blue: databases, green: neighborhood, black: co-expression with 0.400 medium confidence score.

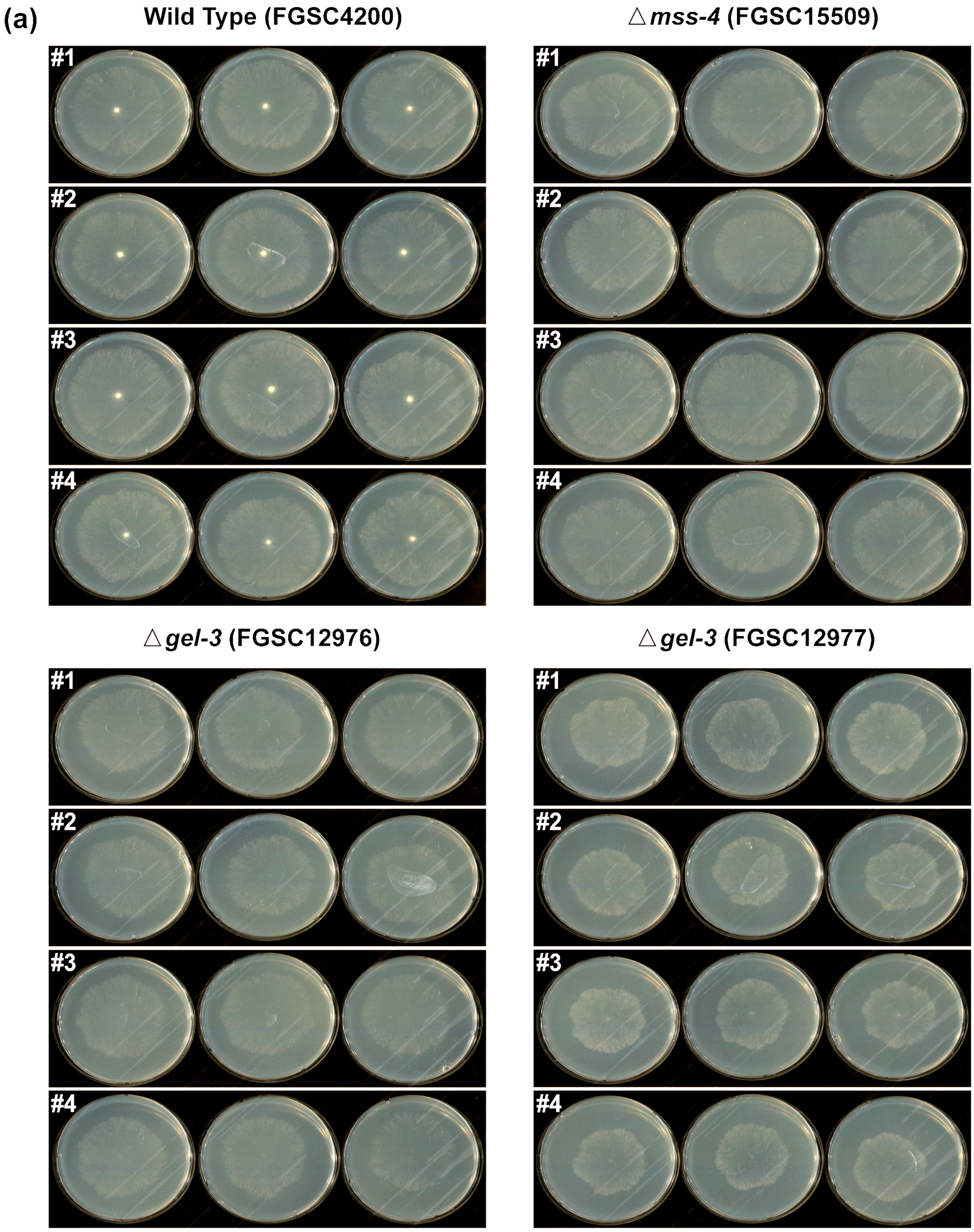
(a)
Wild Type (FGSC4200)
△mss-4 (FGSC15509)
△gel-3 (FGSC12976)
△gel-3 (FGSC12977)
#1
#2
#3
#4

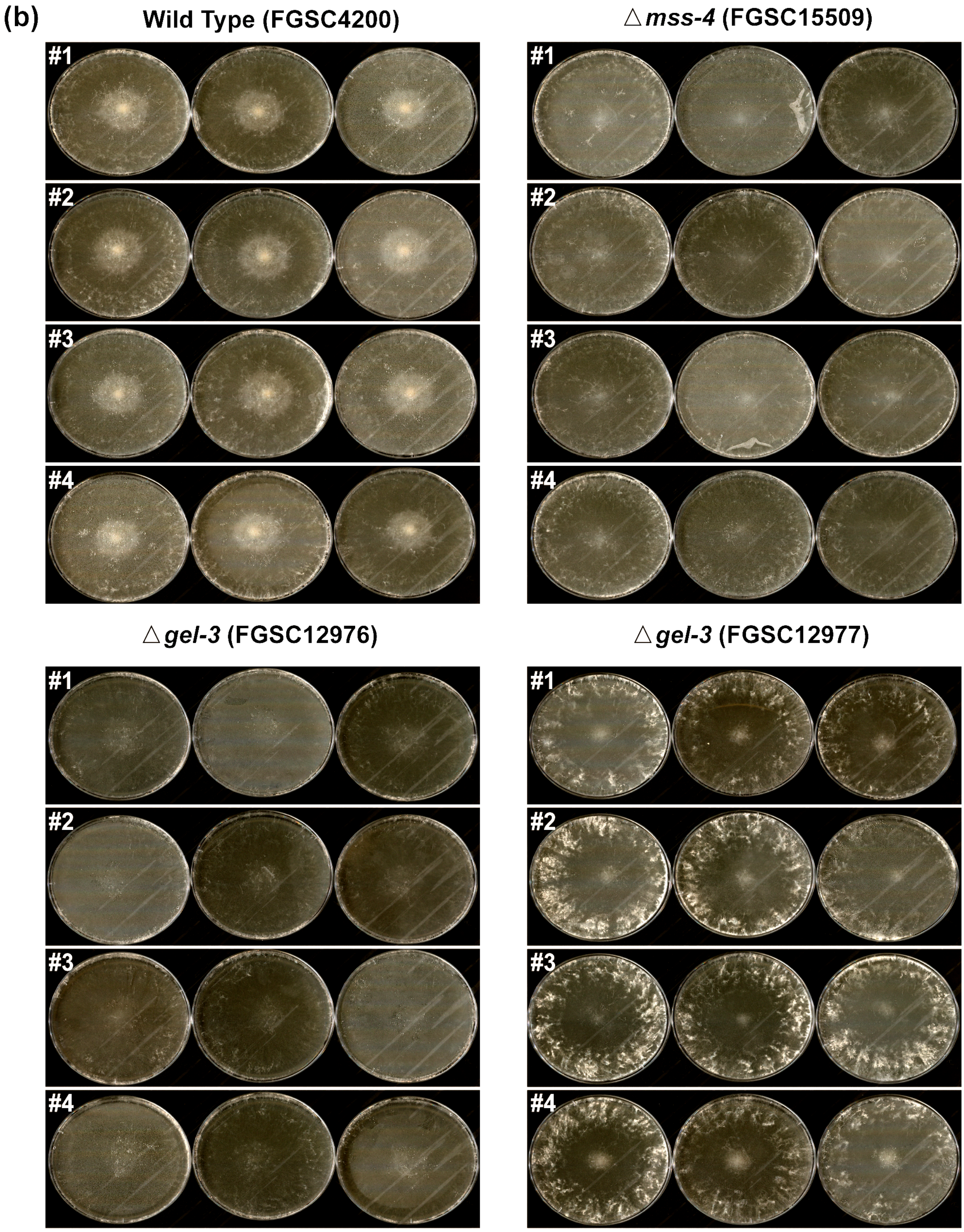
(b)
Wild Type (FGSC4200)
△mss-4 (FGSC15509)
△gel-3 (FGSC12976)
△gel-3 (FGSC12977)
#1
#2
#3
#4

(c)

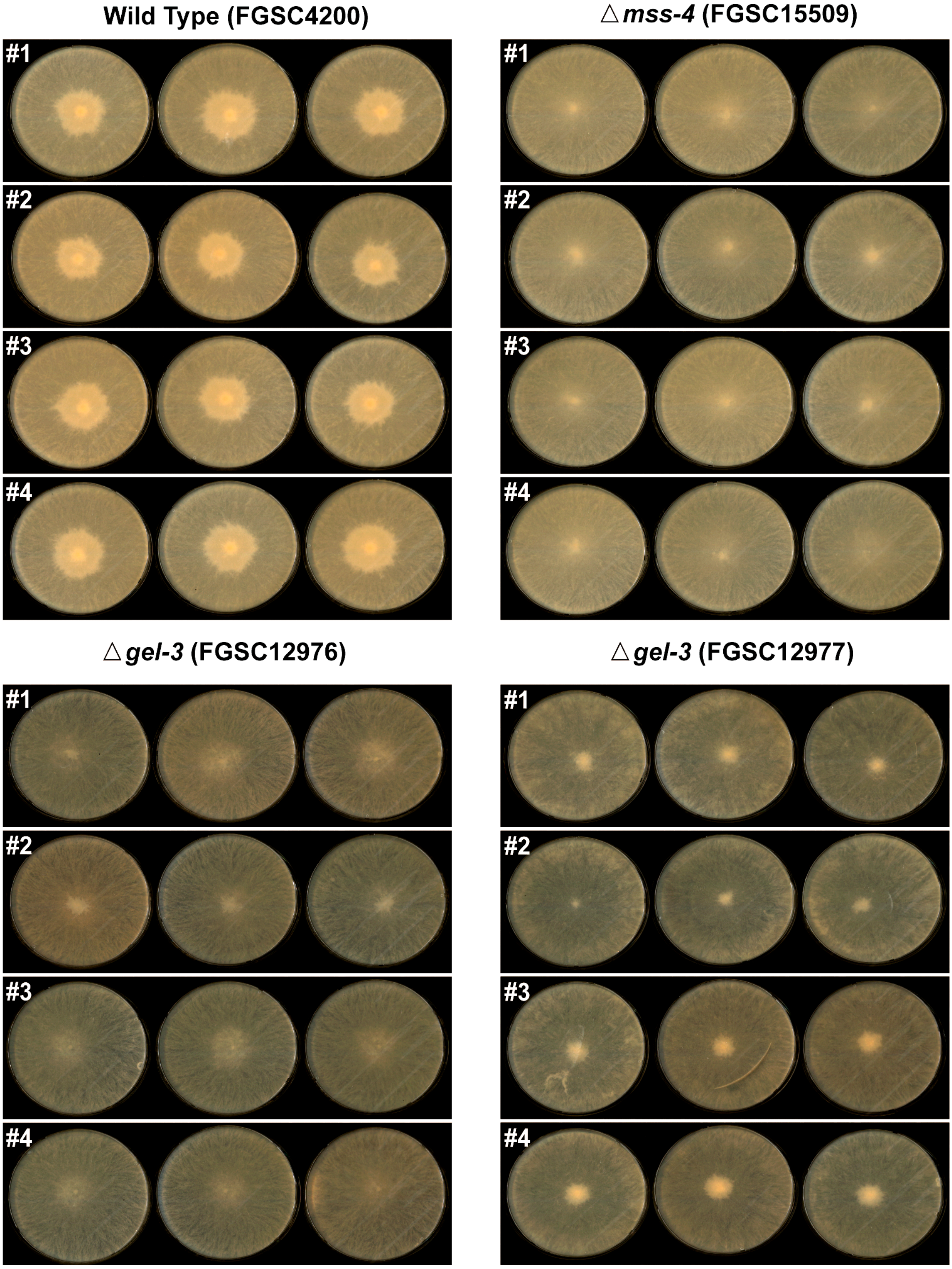
Wild Type (FGSC4200)
△mss-4 (FGSC15509)
△gel-3 (FGSC12976)
△gel-3 (FGSC12977)
#1
#2
#3
#4

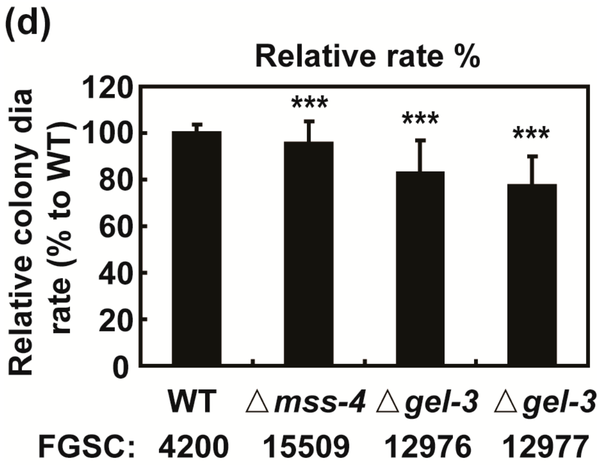


**Supplementary Figure S3. Analysis of basal hyphal growth in *N. crassa*.** Images of all strains (Wild Type/FGSC4200, Δ*mss-4*/FGSC15509, Δ*gel-3*/FGSC12976 and Δ*gel-3*/FG SC12977) grown on plates containing VM agar medium for 24 hours (a) and 48 hours (b, c). (b) and (c) are top and bottom view of plate, respectively. Colony diameters of growing cultures were measured after 24 h and relative growth rates were calculated (d). Twelve replicate plates (three replicates per experiment and four independent experiments) per strain were analyzed.

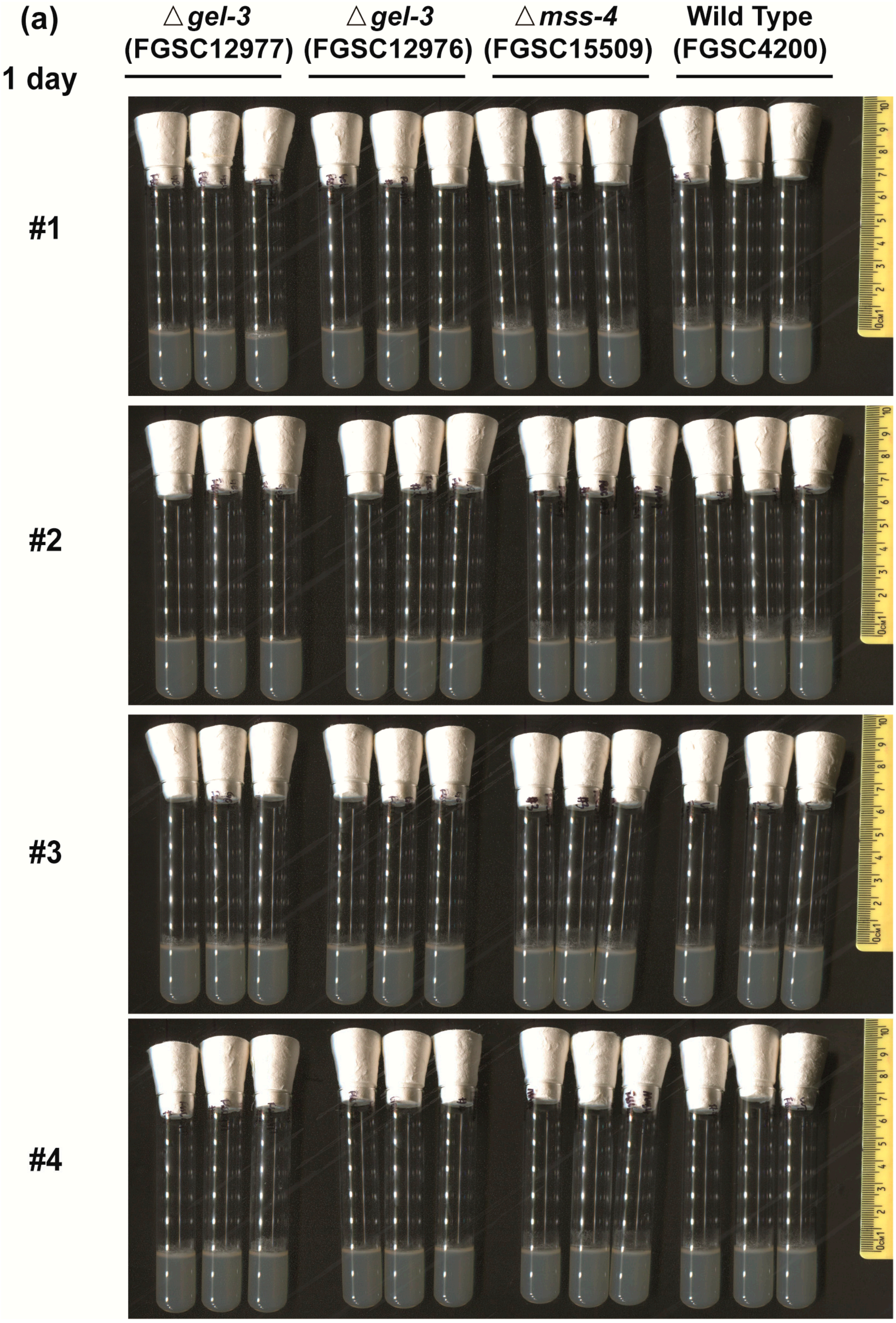
(a)
1 day
△gel-3 (FGSC12977)
△gel-3 (FGSC12976)
△mss-4 (FGSC15509)
Wild Type (FGSC4200)
#1
#2
#3
#4

(b)

2 days

| △*gel-3* (FGSC12977) | △*gel-3* (FGSC12976) | △*mss-4* (FGSC15509) | Wild Type (FGSC4200) |
|---|---|---|---|

#1

#2

#3

#4

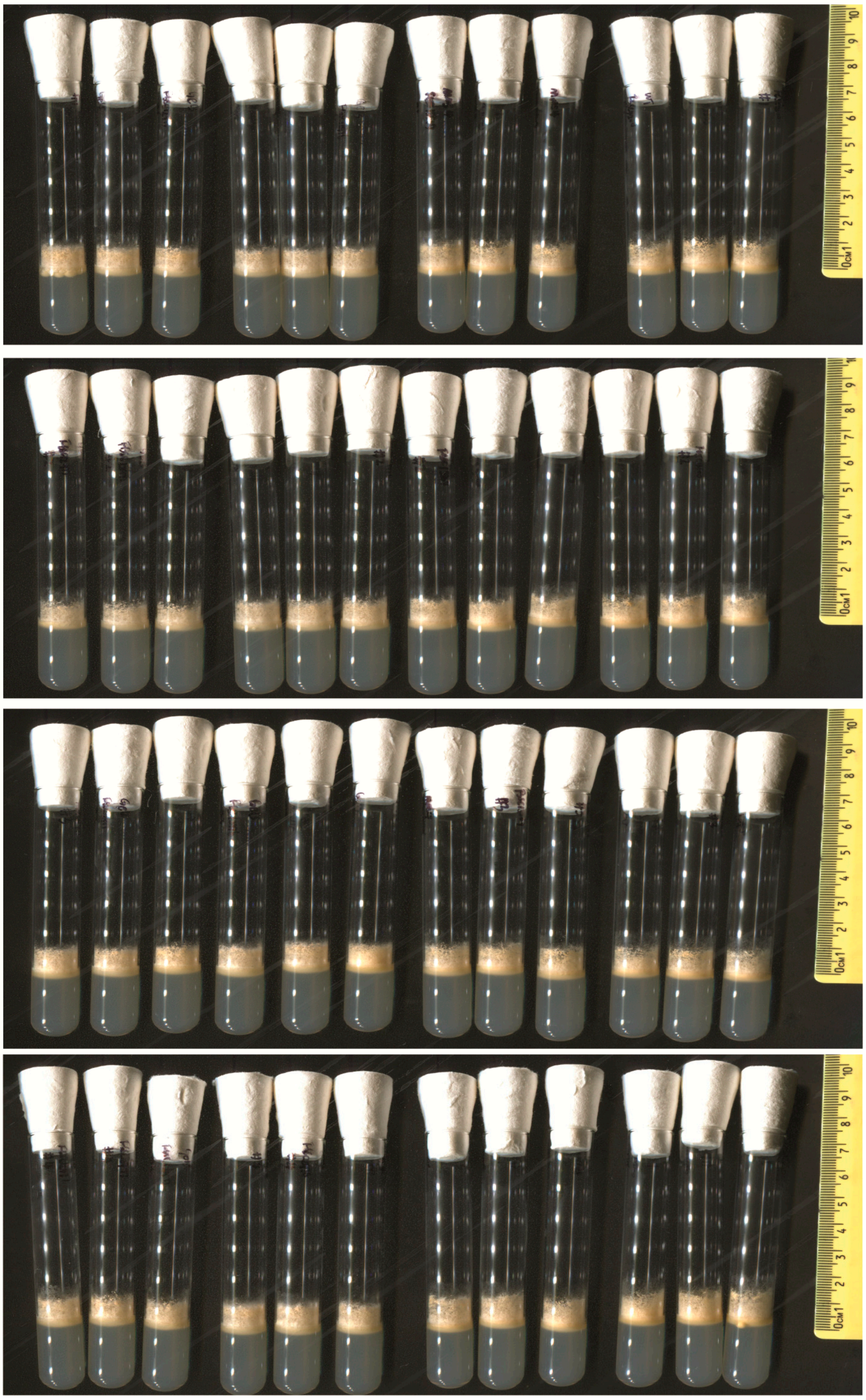

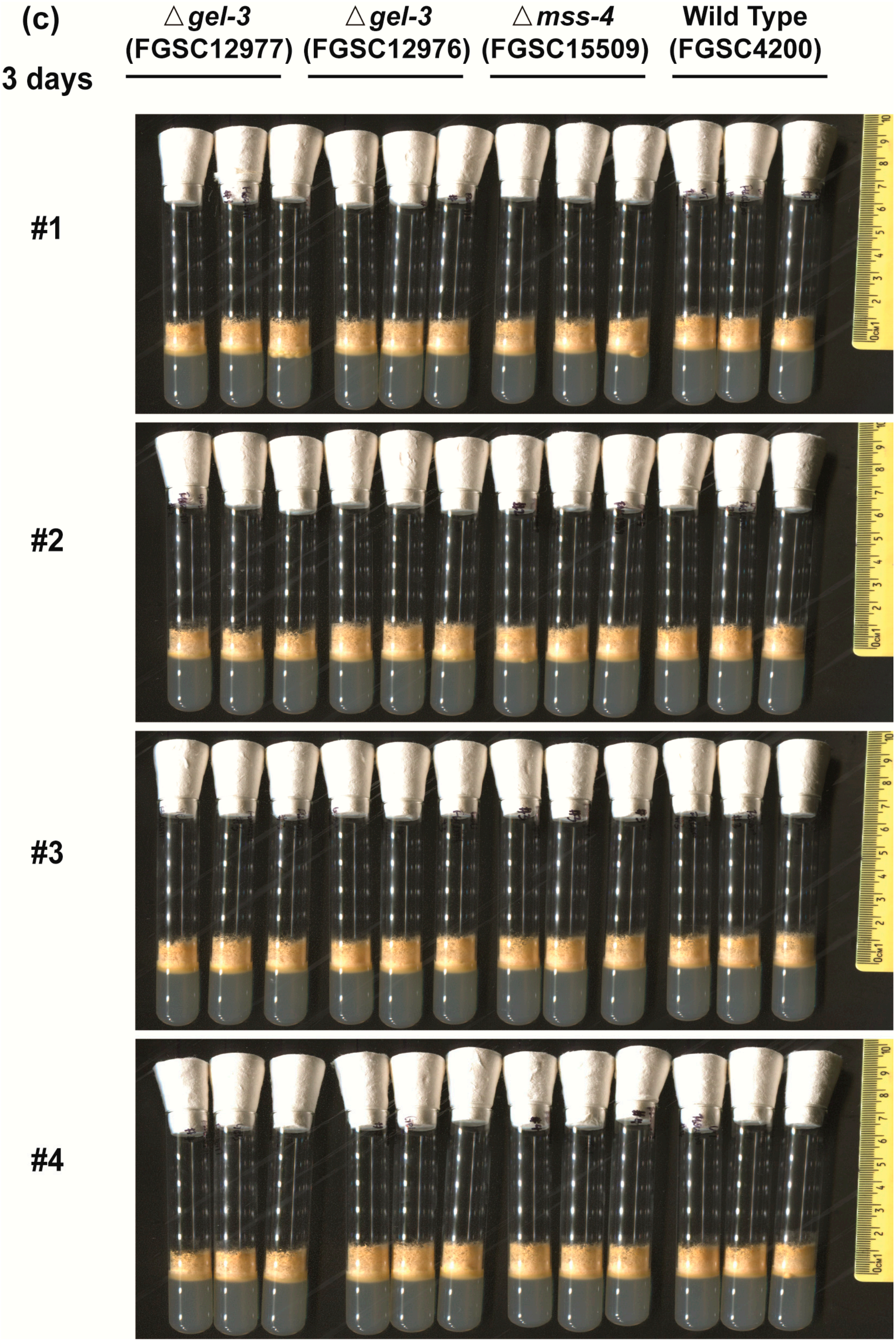
(c)
3 days
△gel-3
(FGSC12977)
△gel-3
(FGSC12976)
△mss-4
(FGSC15509)
Wild Type
(FGSC4200)
#1
#2
#3
#4

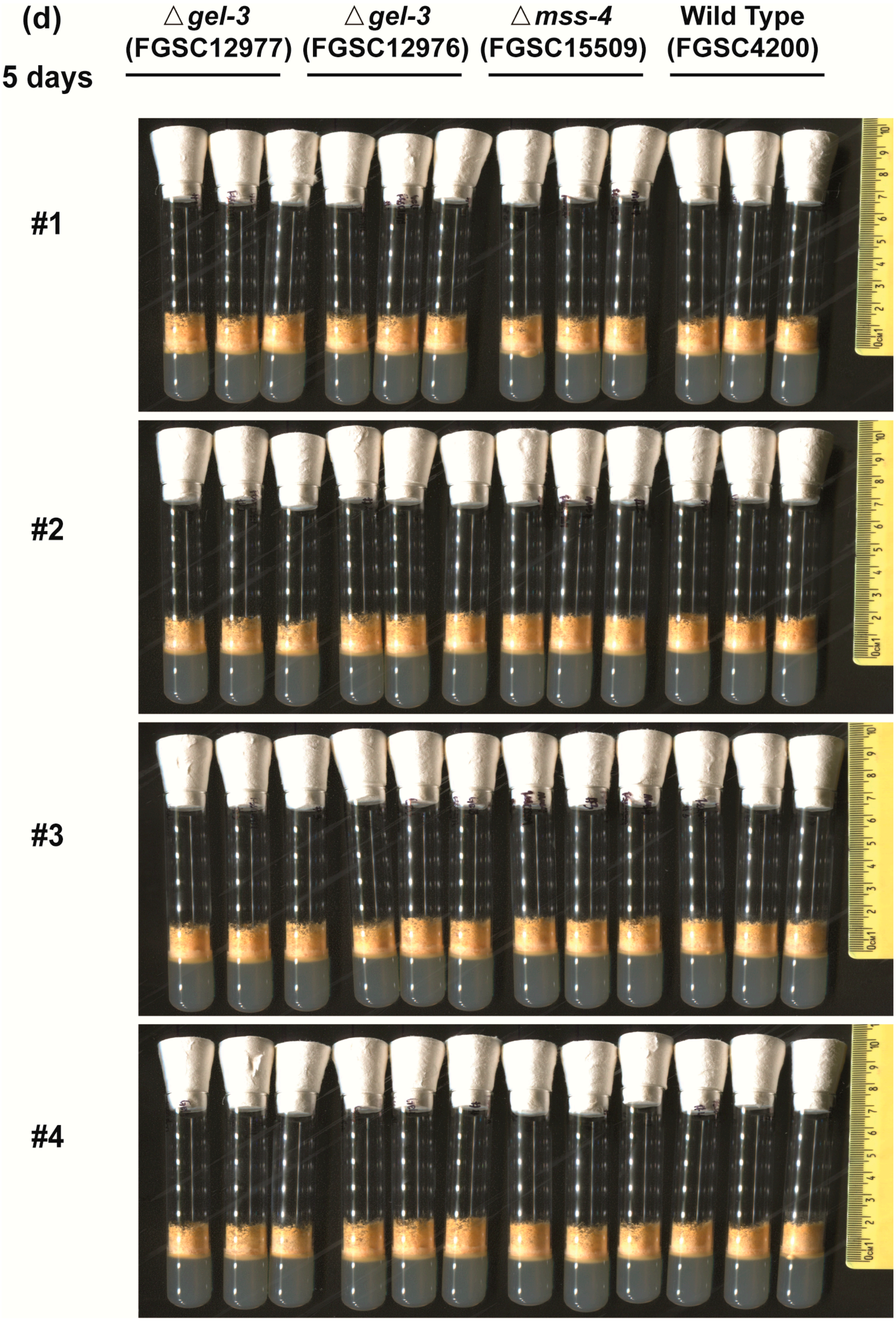
(d)
5 days
△gel-3 (FGSC12977)
△gel-3 (FGSC12976)
△mss-4 (FGSC15509)
Wild Type (FGSC4200)
#1
#2
#3
#4

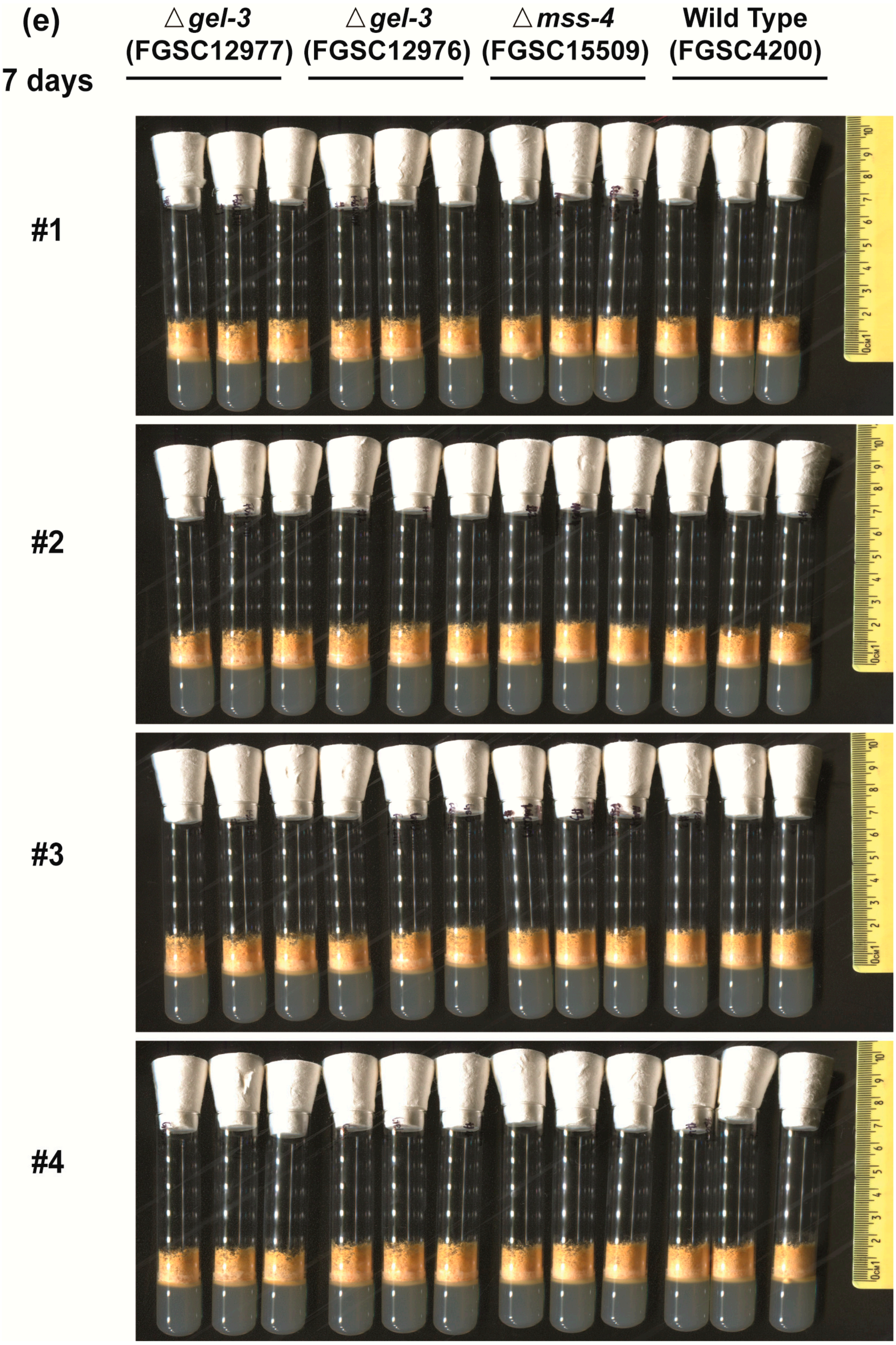


**Supplementary Figure S4. Analysis of aerial hyphal growth and conidia production in *N. crassa*.** Images of all strains (Wild Type/FGSC4200, Δ*mss-4*/FGSC15509, Δ*gel-3*/FGSC 12976 and Δ*gel-3*/FGSC12977) grown in tubes containing VM agar medium for 1 day (a), 2 days (b), 3 days (c), 5 days (d), and 7 days (e). Twelve replicate tubes (three replicates per experiment and four independent experiments) per strain were analyzed.

(a) wild type

#1 #2 #3

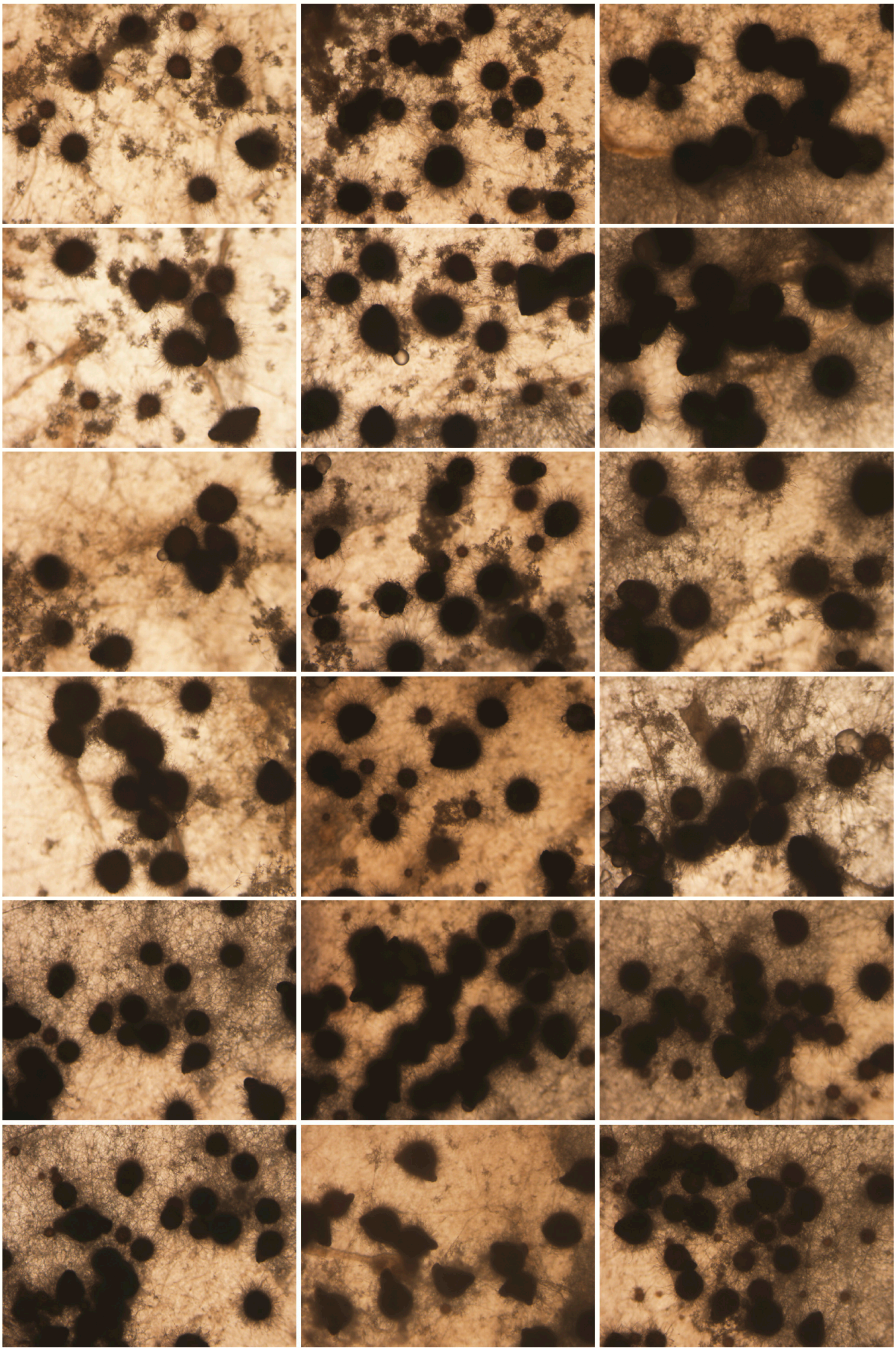

(b) *mss-4* knockout mutant

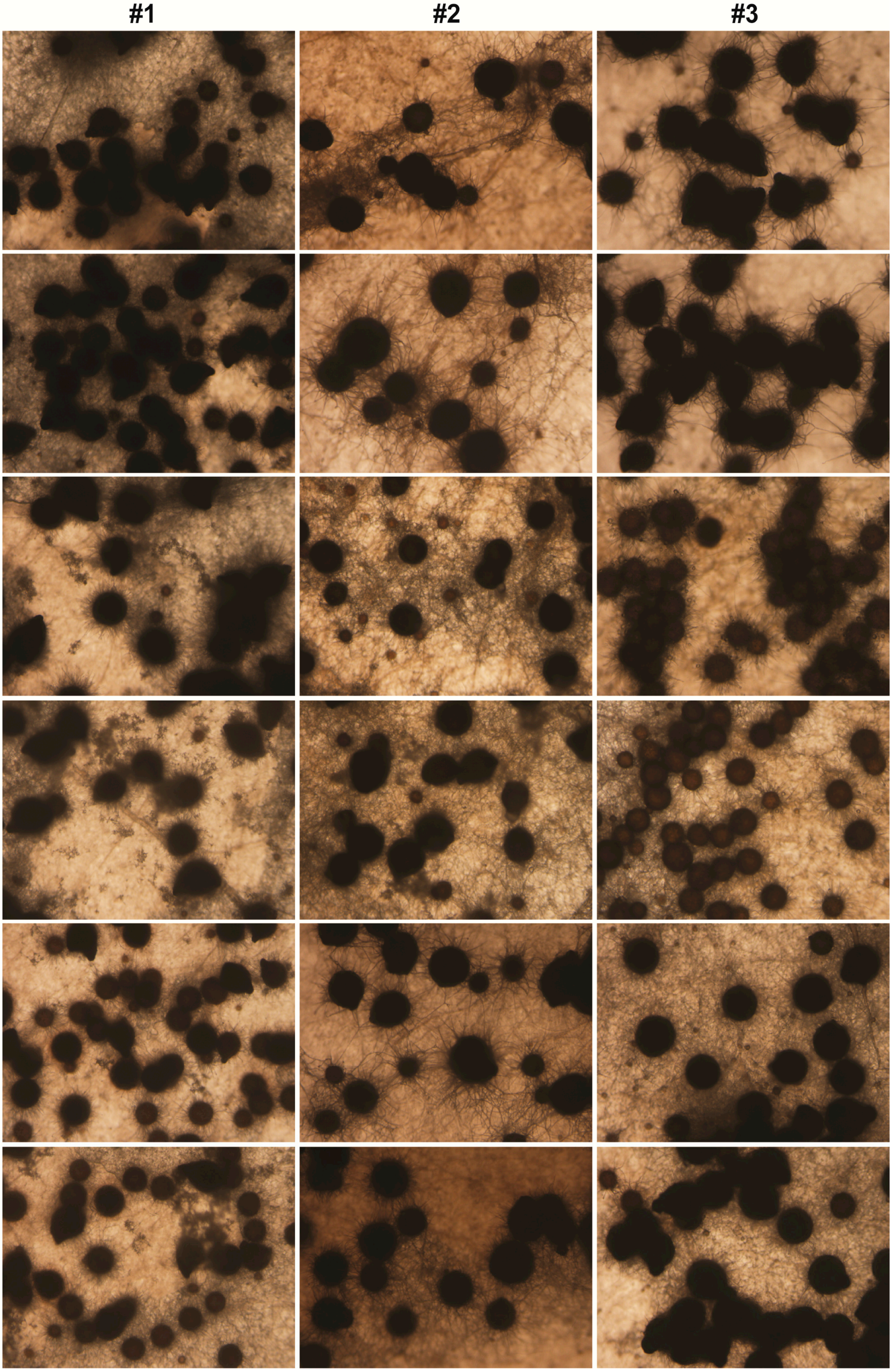

(c) *gel-3* knockout mutant (FGSC12976)

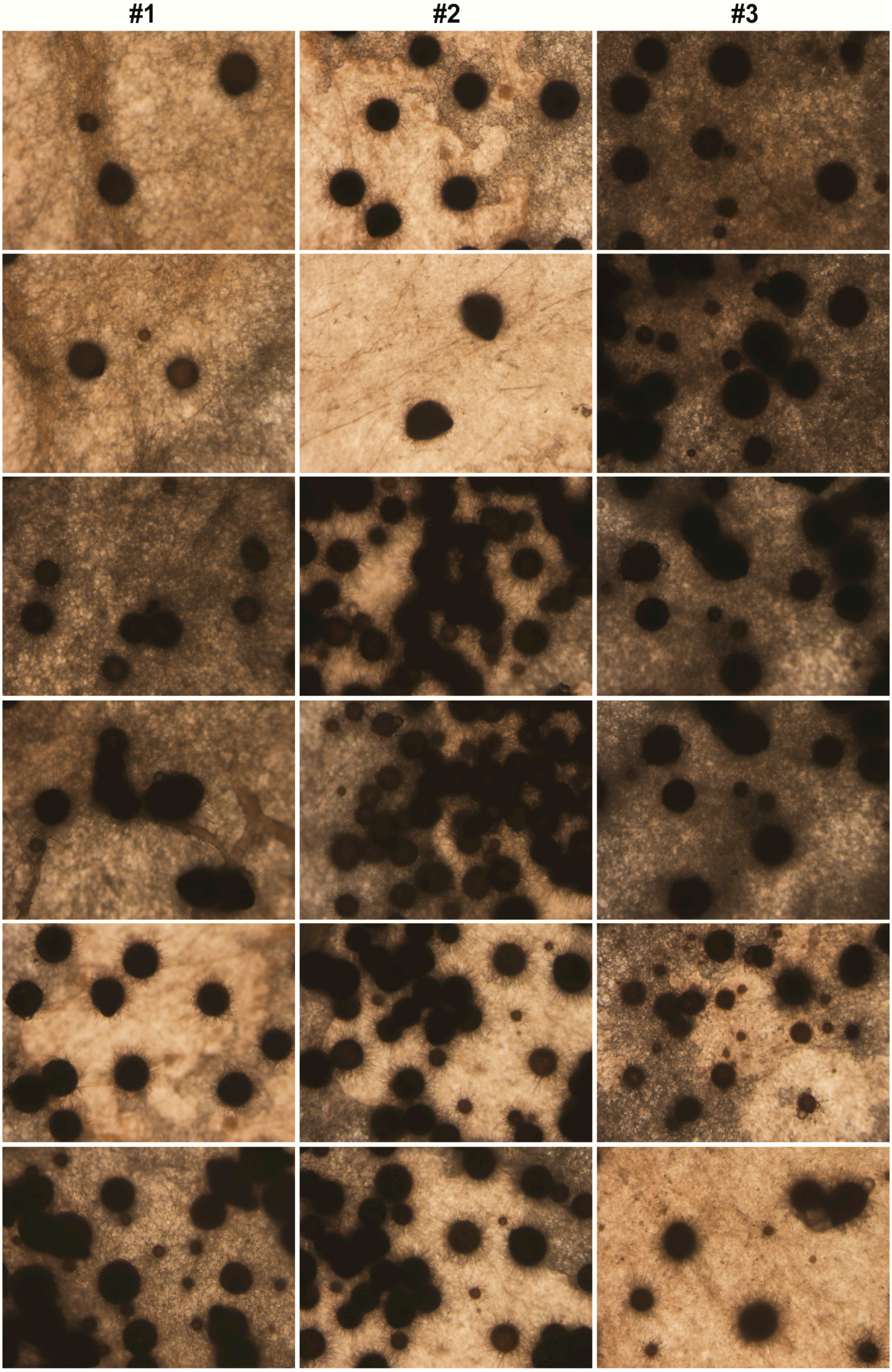

(d) *gel-3* knockout mutant (FGSC12977)

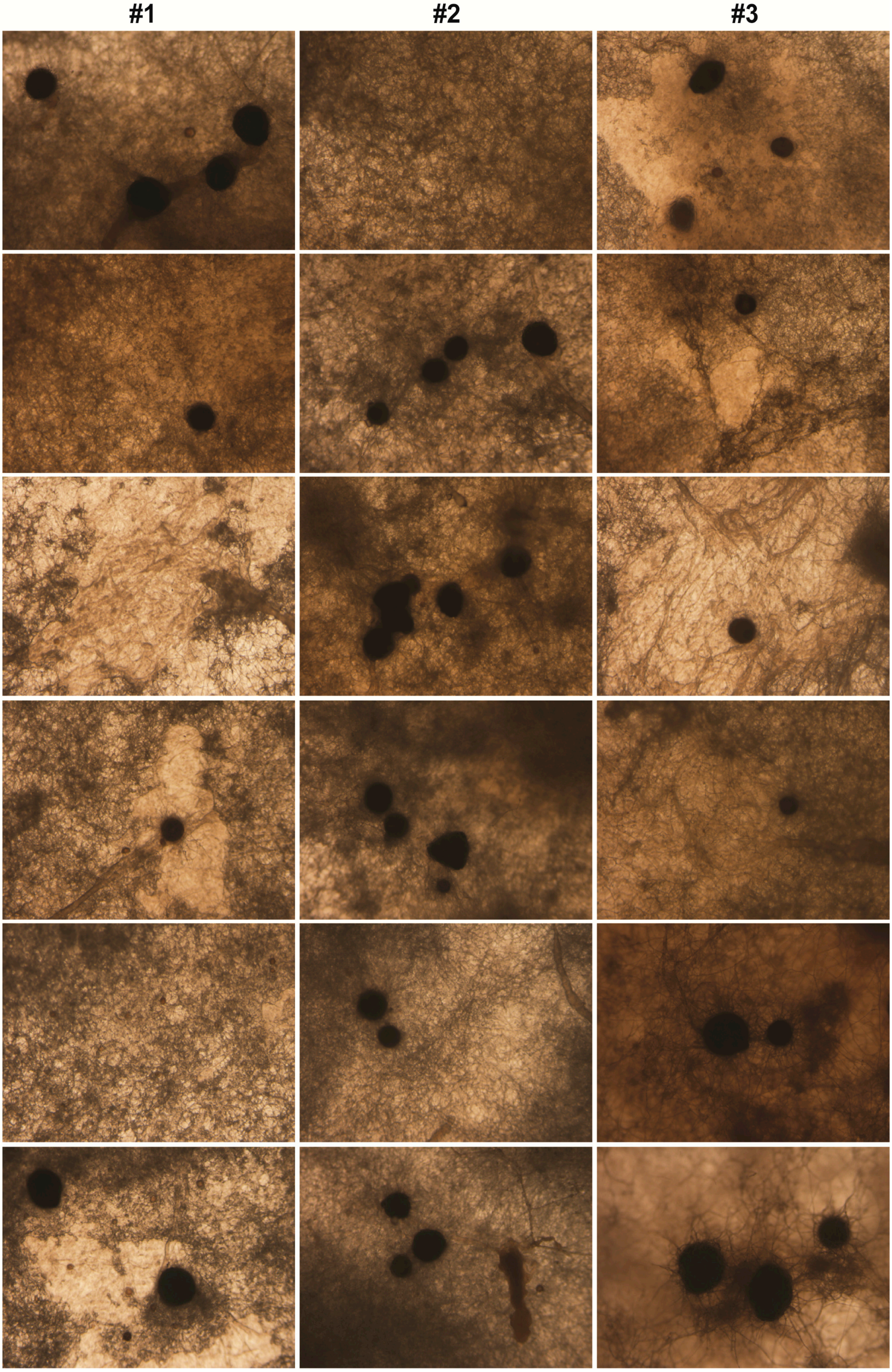

**Supplementary Figure S5. Analysis of perithecia formation in *N. crassa*.** Perithecia formation was analyzed in wild type/FGSC4200 (a), Δ*mss-4*/FGSC15509 (b), Δ*gel-3*/FGSC12976 (c) and Δ*gel-3*/FGSC12977 (d) after fertilized with wile type of opposite mating type. Images were taken 7 days after fertilization. Eighteen replicate images from three independent experiments (three replicate plates per experiment) per strain were analyzed.

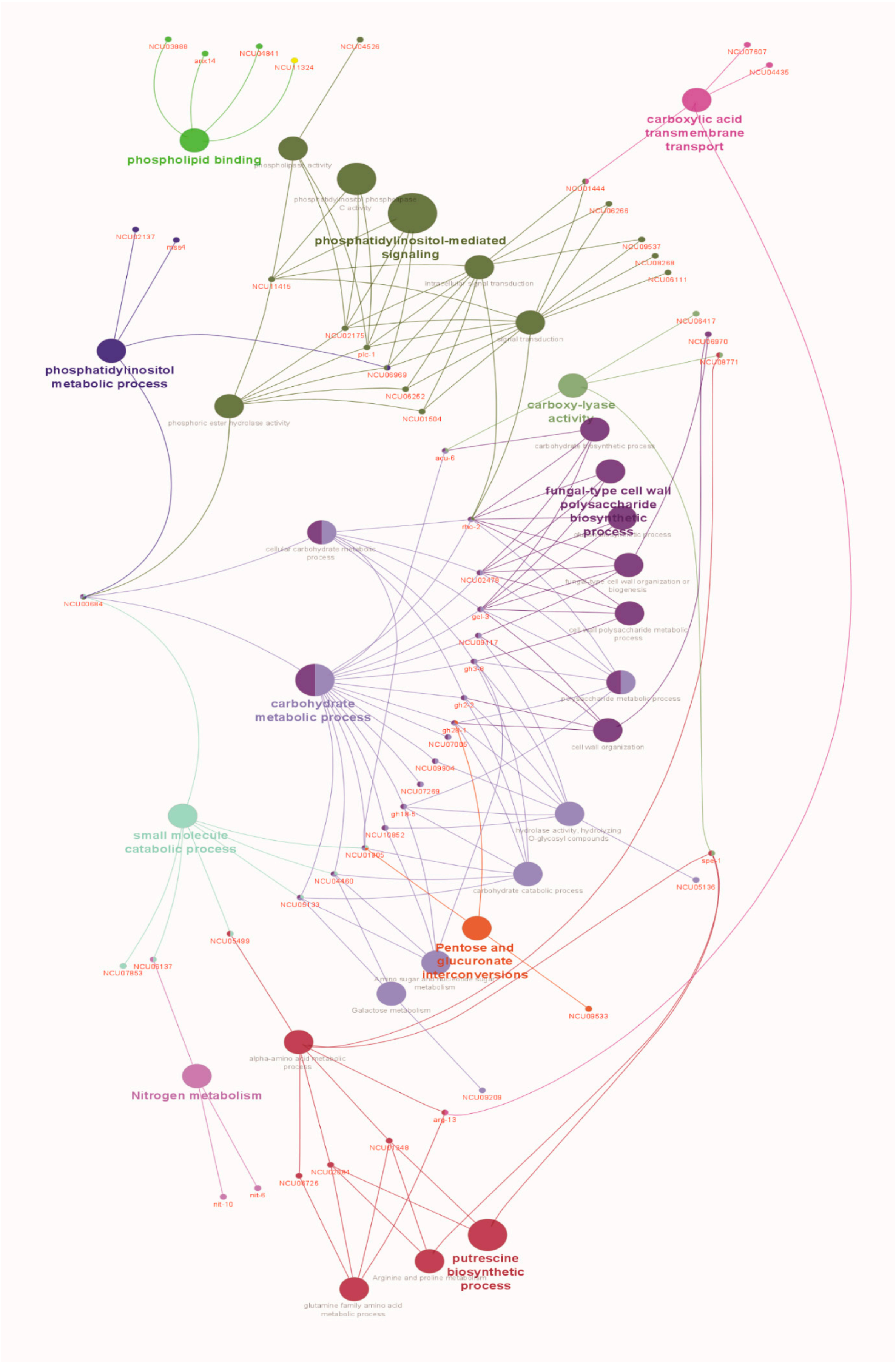
phospholipid binding
NCU03988
NCU04841
NCU11324
NCU04526
phospholipase activity
phosphatidylinositol phospholipase C activity
phosphatidylinositol-mediated signaling
intracellular signal transduction
signal transduction
phosphoric ester hydrolase activity
NCU02137
phosphatidylinositol metabolic process
NCU11415
NCU02175
plc-1
NCU06969
NCU06252
NCU01504
NCU01444
NCU06266
NCU09537
NCU09268
NCU06111
carboxylic acid transmembrane transport
NCU07607
NCU04435
NCU06417
NCU06970
NCU09771
carboxy-lyase activity
carbohydrate biosynthetic process
acu-6
fungal-type cell wall polysaccharide biosynthetic process
rho-2
cellular carbohydrate metabolic process
NCU02478
fungal-type cell wall organization or biogenesis
gel-3
cell wall polysaccharide metabolic process
NCU09117
NCU00684
carbohydrate metabolic process
polysaccharide metabolic process
gh2-2
cell wall organization
NCU07005
NCU09904
NCU07269
gh18-5
NCU10852
NCU01905
hydrolase activity, hydrolyzing O-glycosyl compounds
small molecule catabolic process
NCU04460
NCU05133
carbohydrate catabolic process
spe-1
NCU05136
NCU05499
NCU06137
NCU07853
Pentose and glucuronate interconversions
Amino sugar and nucleotide sugar metabolism
Galactose metabolism
NCU09533
alpha-amino acid metabolic process
Nitrogen metabolism
NCU09209
arg-13
NCU01948
NCU06726
nit-10
nit-6
putrescine biosynthetic process
Arginine and proline metabolism
glutamine family amino acid metabolic process

**Supplementary Figure S6. GO terms and KEGG pathway visualized using the ClueGO/CluePedia plugin from Cytoscape.** Significant GO and KEGG terms are presented together with specific gene interactions. Nodes of the same color represent a functional group. The gene names involved in each group are shown in red. The maximum node (minimum *p*-value) in the same functional group is shown in bold and was also considered the most abundant term. $p \leq 0.05$, Benjamini-Hochberg correction, and kappa score $\geq 0.4$ were used as criteria for ClueGO analysis.
